# Mystery of fatal ‘staggering disease’ unravelled: novel rustrela virus causes severe meningoencephalomyelitis in domestic cats

**DOI:** 10.1038/s41467-023-36204-w

**Published:** 2023-02-04

**Authors:** Kaspar Matiasek, Florian Pfaff, Herbert Weissenböck, Claudia Wylezich, Jolanta Kolodziejek, Sofia Tengstrand, Frauke Ecke, Sina Nippert, Philip Starcky, Benedikt Litz, Jasmin Nessler, Peter Wohlsein, Christina Baumbach, Lars Mundhenk, Andrea Aebischer, Sven Reiche, Pia Weidinger, Karin M. Olofsson, Cecilia Rohdin, Christiane Weissenbacher-Lang, Julia Matt, Marco Rosati, Thomas Flegel, Birger Hörnfeldt, Dirk Höper, Rainer G. Ulrich, Norbert Nowotny, Martin Beer, Cecilia Ley, Dennis Rubbenstroth

**Affiliations:** 1 Section of Clinical & Comparative Neuropathology, Institute of Veterinary Pathology, Centre for Clinical Veterinary Medicine, Ludwig-Maximilians-Universitaet-Muenchen, Munich, Germany; 2 Institute of Diagnostic Virology, Friedrich-Loeffler-Institut, Greifswald-Insel Riems, Germany; 3 Institute of Pathology, University of Veterinary Medicine Vienna, Vienna, Austria; 4 Institute of Virology, University of Veterinary Medicine Vienna, Vienna, Austria; 5 Department of Biomedical Sciences and Veterinary Public Health, Swedish University of Agricultural Sciences (SLU), Uppsala, Sweden; 6 Department of Wildlife, Fish, and Environmental Studies, Swedish University of Agricultural Sciences (SLU), Umeå, Sweden; 7 Organismal and Evolutionary Biology Research Programme, Faculty of Biological and Environmental Sciences, University of Helsinki, Helsinki, Finland; 8 Institute of Novel and Emerging Infectious Diseases, Friedrich-Loeffler-Institut, Greifswald-Insel Riems, Germany; 9 Department of Small Animal Medicine and Surgery, University of Veterinary Medicine Hannover, Hannover, Germany; 10 Department of Pathology, University of Veterinary Medicine Hannover, Hannover, Germany; 11 State Office for Agriculture, Food Safety and Fisheries, Rostock, Germany; 12 Institute of Veterinary Pathology, Freie Universität Berlin, Berlin, Germany; 13 Department of Experimental Animal Facilities and Biorisk Management, Friedrich-Loeffler-Institut, Greifswald-Insel Riems, Germany; 14 Department of Pathology and Wildlife Diseases, National Veterinary Institute (SVA), Uppsala, Sweden; 15 Department of Clinical Sciences, Swedish University of Agricultural Sciences (SLU), Uppsala, Sweden; 16 Anicura, Albano Small Animal Hospital, Danderyd, Sweden; 17 Department of Small Animal Medicine, Leipzig University, Leipzig, Germany; 18 German Centre for Infection Research (DZIF), Partner site Hamburg-Lübeck-Borstel-Riems, Greifswald-Insel Riems, Germany; 19 College of Medicine, Mohammed Bin Rashid University of Medicine and Health Sciences, Dubai, United Arab Emirates

**Keywords:** Virology, Neurological disorders

## Abstract

‘Staggering disease’ is a neurological disease entity considered a threat to European domestic cats (*Felis catus*) for almost five decades. However, its aetiology has remained obscure. Rustrela virus (RusV), a relative of rubella virus, has recently been shown to be associated with encephalitis in a broad range of mammalian hosts. Here, we report the detection of RusV RNA and antigen by metagenomic sequencing, RT-qPCR, in-situ hybridization and immunohistochemistry in brain tissues of 27 out of 29 cats with non-suppurative meningoencephalomyelitis and clinical signs compatible with’staggering disease’ from Sweden, Austria, and Germany, but not in non-affected control cats. Screening of possible reservoir hosts in Sweden revealed RusV infection in wood mice (*Apodemus sylvaticus*). Our work indicates that RusV is the long-sought cause of feline ‘staggering disease’. Given its reported broad host spectrum and considerable geographic range, RusV may be the aetiological agent of neuropathologies in further mammals, possibly even including humans.

## Abstract (web summary)

Rustrela virus (RusV) was detected in the brains of 27 out of 29 domestic cats with ‘staggering disease’, but not of 29 control cats. This suggests RusV as the long-sought causative agent of ‘staggering disease’, which had been obscure for 50 years.

## Introduction

Throughout mammalian species, inflammatory disorders of the central nervous system (CNS) are associated with substantial morbidity, mortality and long-term neurological deficits. Aetiopathogenically, they can be broadly categorised into infectious and immune-mediated disorders^1^. All too often, however, the cause of an encephalitis remains unknown and leaves clinicians, patients and owners of affected pets with considerable uncertainty about its origin, treatment options and, hence, prognosis. The latter holds true particularly for the large histopathologically convergent group of non-suppurative, lymphohistiocytic encephalitides. A substantial proportion of these cases remains unsolved using conventional diagnostic methods, such as immunohistochemistry (IHC), in situ hybridization (ISH), and polymerase chain reaction (PCR) techniques for regional neurotropic pathogens^2–9^.

One of those controversial encephalitides of possibly infectious origin is the so-called ‘staggering disease’ of domestic cats (*Felis catus*). It has been described first in the 1970s in the Swedish Lake Mälaren region between Stockholm and Uppsala^10^, which remains a hotspot of ‘staggering disease’ to the present. In the 1990s, feline ‘staggering disease’ was also described in a region close to Vienna in Austria^11,12^. Neurologic disorders resembling this disease entity have been described also in domestic cats in other European countries^6,13,14^ and even in other felids^15,16^.

The most prototypic clinical sign of ‘staggering disease’ is hind leg ataxia with a generally increased muscle tone resulting in a staggering gait. In addition, a broad range of other neurologic signs may occur, including the inability to retract the claws, hyperaesthesia and occasionally tremors and seizures. Behavioural alterations include enhanced vocalization, depression, becoming more affectionate, and rarely aggression^10,12,17,18^. The disease progression usually lasts a few days to a few weeks, but may also continue for more than a year, and it generally results in deterioration requiring euthanasia for animal welfare reasons. The histopathology of ‘staggering disease’ is characterized by a non-suppurative, predominantly lymphohistiocytic meningoencephalomyelitis with angiocentric immune cell infiltration and perivascular cuffing predominantly in the grey matter of the CNS^10,12,17–19^.

Due to its usually typical clinical presentation, its uniform histopathology and its geographically associated occurrence, feline ‘staggering disease’ has always been suspected as a cohesive disease entity with a consistent aetiology. While the microscopic pattern has suggested a viral origin, its aetiological agent has remained undetermined for almost five decades. For a long time, Borna disease virus 1 (BoDV-1; species *Orthobornavirus bornaense*; family *Bornaviridae*), which causes neurologic disorders in various mammals including humans^20–22^, has spearheaded the panel of aetiological candidates^19,23–28^. BoDV-1-induced neurologic disease of domestic cats has been demonstrated after experimental infection^29^ as well as in a single case of confirmed natural infection in Switzerland^30^. However, unequivocally confirmed BoDV-1 infections in domestic mammals are reported only from restricted endemic areas in Germany, Austria, Switzerland and Liechtenstein^20–22,31,32^, whereas results suggesting natural BoDV-1 infections in cats with ‘staggering disease’ in Sweden remained inconclusive. In particular, unambiguous and consistent BoDV-1 detection with independent diagnostic methods could not be presented. Furthermore, BoDV-1 sequences reported from Sweden did not match the phylogeographic pattern, as observed for evidently infected individuals in the known endemic areas of BoDV-1, and are therefore suspected to rather represent laboratory artifacts^20,31,32^.

Fortunately, advances in clinical metagenomics over the previous years have provided us with promising tools for the detection of new or unexpected pathogens involved in hitherto unexplained encephalitides^33–38^.

One of these recently discovered encephalitic agents is rustrela virus (RusV; *Rubivirus strelense; Matonaviridae*), a relative of rubella virus (RuV; *Rubivirus rubellae*), the causative agent of rubella in humans^37,39^. RusV was first identified in the brains of various mammals in a zoo close to the Baltic Sea in northern Germany^37,40^. These animals had suffered from neurologic disorders associated with lymphohistiocytic encephalitis^37,38,40^. Yellow-necked field mice (*Apodemus flavicollis*) without apparent encephalitis were considered as possible reservoir hosts of the virus in that area^37,40^.

Here, we report the presence of RusV in the brains of cats with non-suppurative, lymphohistiocytic meningoencephalomyelitis and neurologic disorders matching the description of ‘staggering disease’ from Sweden, Austria, and Germany. The virus was first detected by application of an established metagenomic workflow^41^, which was further confirmed by independent methods, including RT-qPCR, ISH and IHC. In contrast, RusV was not detected in the brains of control cats without neurologic disease or with encephalopathies of other causes from the same or nearby geographic regions. Thus, our investigation on recent and historic cases, dating back to the 1990s, provide evidence that RusV has been the causative agent of long-known feline ‘staggering disease’.

## Results

### Failure to detect BoDV-1 infection in cats with ‘staggering disease’

In an attempt to investigate the aetiology of ‘staggering disease’, we assembled frozen or formalin-fixed paraffin-embedded (FFPE) brain samples from 29 cats with multifocal lymphohistiocytic meningoencephalomyelitis and a clinical presentation consistent with the description of ‘staggering disease’, particularly ataxia and other gait disturbances. The majority of the cats originated from the previously identified hotspots of ‘staggering disease’ in Sweden (*n* = 15) and Austria (*n* = 9) and had been diagnosed by veterinarians experienced in the phenomenology of ‘staggering disease’. Five additional cases matching the same inclusion criteria originated from different regions in Germany. Brain tissues of these 29 cats were examined for the presence of bornaviruses by RT-qPCR assays detecting the RNA of BoDV-1 and other orthobornaviruses^34^ (Supplementary Table 1), and by IHC using a monoclonal antibody targeting the BoDV-1 nucleoprotein (Supplementary Fig. 1). Neither bornavirus RNA nor antigen were detected.

### RusV sequences identified in cats with ‘staggering disease’ by metagenomic analysis

**Fig. 1:**
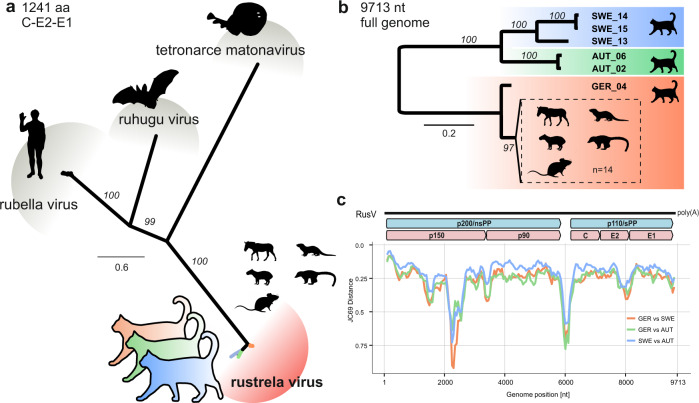
Sequence comparison of complete rustrela virus (RusV) genome sequences from cats from Sweden, Austria, and Germany. **a** The amino acid sequences of the structural polyprotein (p110/sPP) of all known matonaviruses were aligned and a maximum-likelihood (ML) phylogenetic tree was calculated (IQ-TREE2 version 2.2.0; FLU + F + I + G4; 100,000 ultrafast bootstraps). Bootstrap support values are shown in italics. **b** ML tree of complete or nearly complete RusV genome sequences from cats with ‘staggering disease’ and all publicly available RusV sequences (IQ-TREE2 version 2.2.0; TIM3 + F + I; 100,000 ultrafast bootstraps). Sequences from Sweden, Austria, and Germany are highlighted in blue, green, and orange, respectively. Sequences from a previously identified German RusV cluster from zoo animals with encephalitis and apparently healthy yellow-necked field mice (*Apodemus flavicollis*)^37,38,40^ are presented in a dashed box. Bootstrap support values are shown at the nodes. **c** The genetic variability of RusV lineages from Sweden, Austria, and Germany is presented as mean pairwise JC69 distance using a sliding window analysis (window: 200 nt; step size: 50 nt). The genomic organization of RusV is shown, highlighting the non-structural (p200/nsPP) and structural (p110/sPP) polyprotein open reading frames, as well as the mature cleavage products protease (p150), RNA-directed RNA polymerase (p90), capsid protein (C), and glycoproteins E2 and E1.

Selected samples were subsequently analysed using a generic metagenomic sequencing workflow^41^. In an initial analysis using blastx, sequence reads with the highest identity to RusV were identified in 15 out of 17 tested samples from these three countries (Table 1). Additional high-throughput sequencing (HTS), assisted by target enrichment using the panRubi myBaits sets v2 and v3, resulted in complete RusV genome sequences for three cats from Sweden (animals SWE_13, SWE_14 and SWE_15) and one cat from northeastern Germany (GER_04), as well as a complete and an almost complete genome sequence for two cats from Austria (AUT_02 and AUT_06, respectively). The newly identified RusV sequences clearly clustered with other RusV sequences when compared to related matonaviruses (Fig. 1a), based on amino acid (aa) sequences of the structural polyprotein (p110/sPP). The genome nucleotide (nt) sequences from Austria and Sweden formed separate phylogenetic lineages in comparison to the sequences from Germany (Fig. 1b). While sequence GER_04 possessed at least 92.1% nt sequence identity with the previously published German RusV sequences, the minimum nt identities of the Swedish and Austrian sequences to the German sequences were only 76.7% and 76.0%, respectively, but 80.7% to each other (Supplementary Fig. 2). The genome organization of the newly discovered RusV sequences (Fig. 1c) was consistent with those of previously published RusV genomes^37,40^. Using a sliding window analysis, we identified a highly conserved region at the 5’ terminus of the RusV genomes (approximate positions 1 to 300). Regions of particularly high variability covered the intergenic region between the p200 and p110 open reading frames (ORF) as well as a stretch of the p150-encoding sequence around nt positions 2100–2600 (Fig. 1c).

**Table 1:** Rustrela virus (RusV) detection in brain samples from cats with or without signs of ‘staggering disease’

Group	Years	*n*	RusV detection (positive/total animals)				
Country			HTS	RT-qPCR	ISH	IHC	Total^a^
**Cats matching the criteria of ‘staggering disease’**							
Sweden	2017–2021	15	9/9	15/15	15/15	15/15	15/15
Austria	1991–1993	9	4/5	8/9	7/9	8/9	8/9
Germany	2017–2022	5	2/3	3/5	2/5	4/5	4/5
**Cats with other types of encephalitis**^b^							
Germany	2017–2020	8	0/3	0/8	0/3	0/8	0/8
**Cats without encephalitis**^b^							
Sweden	2021–2022	7	n.d.	0/7	0/6	0/7	0/7
Austria	2021	5	n.d.	0/5	0/5	0/5	0/5
Germany	2018–2020	9	n.d.	0/9	0/3	0/9	0/9

*HTS* high-throughput sequencing followed by metagenomic analysis, *ISH* in situ hybridization using RNAscope, *IHC* immunohistochemistry, *n.d.* not determined.

^a^Cats were considered RusV-positive if RusV RNA and/or antigen was detected by at least two of the applied methods (RT-qPCR, ISH, IHC, sequencing by HTS).

^b^Detailed information on individuals included in these groups is provided in Supplementary Table 4.

### Detection of RusV RNA using a novel broadly reactive panRusV RT-qPCR assay

**Fig. 2:**
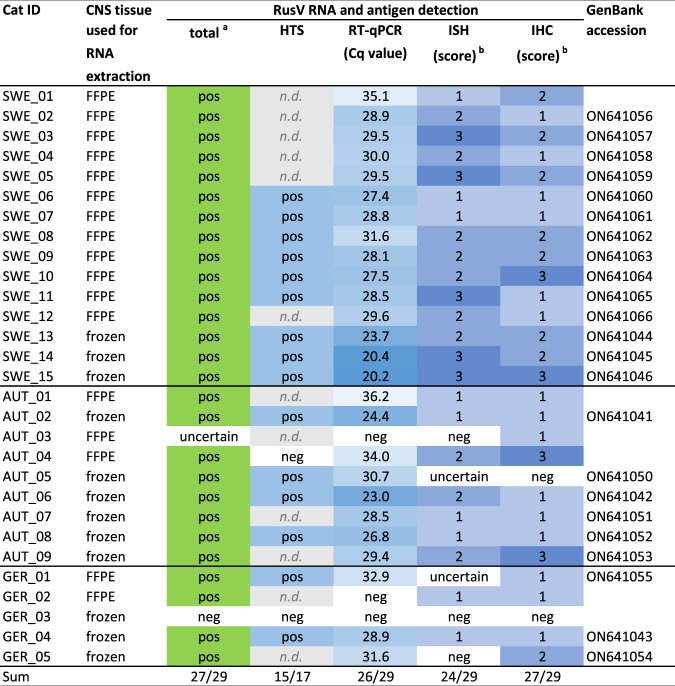
Detection of rustrela virus (RusV) RNA and antigen in cats with ‘staggering disease’. Cats with clinical signs and histopathological lesions consistent with the criteria of ‘staggering disease’ were tested by different independent diagnostic methods. Cats were considered RusV-positive if RusV RNA and/or antigen was detected by at least two of the applied methods (RT-qPCR, ISH, IHC, sequencing by HTS). Cats with a positive result in only one assay were considered as uncertain. Green indicates an overall positive result of an individual, whereas blue indicates positivity for the detection of RusV RNA or antigen in an individual test. The intensity of the colour semiquantitatively reflects the strength of the signal of the respective assay. The scoring of IHC and RNAscope ISH signals is described in Table 2. CNS central nervous system, FFPE formalin-fixed paraffin-embedded, HTS high-throughput sequencing followed by metagenomic analysis, Cq cycle of quantification, ISH in situ hybridization using RNAscope, IHC immunohistochemistry, pos positive, neg negative, n.d. not determined.

Since the initially published RT-qPCR assay RusV Mix-1^37^ was unable to detect RusV RNA in samples from Sweden and Austria (data not shown), we designed a new set of primers and probe targeting the highly conserved region at the 5’ end of the genome (Supplementary Fig. 3; Supplementary Table 1). This newly established panRusV assay readily detected RusV RNA in the brains of all 15 Swedish cats with ‘staggering disease’, eight out of nine Austrian cats^11,12^, and three out of five cats from Germany (Table 1). Results were moderately to strongly positive for frozen tissue (cycle of quantification [Cq] values 20 to 32), and rather weakly positive for animals of which only FFPE material was available (Cq 27 to 36; Fig. 2; Supplementary Fig. 4a). In contrast, RusV RNA was not detected in frozen brain samples from 21 control cats without encephalitis originating from Sweden, Austria, and Germany, or in eight cats from Germany suffering from other types of encephalitis (Table 1; Supplementary Table 4).

### Detection of RusV RNA and antigen in neural tissue by ISH and IHC

**Fig. 3:**
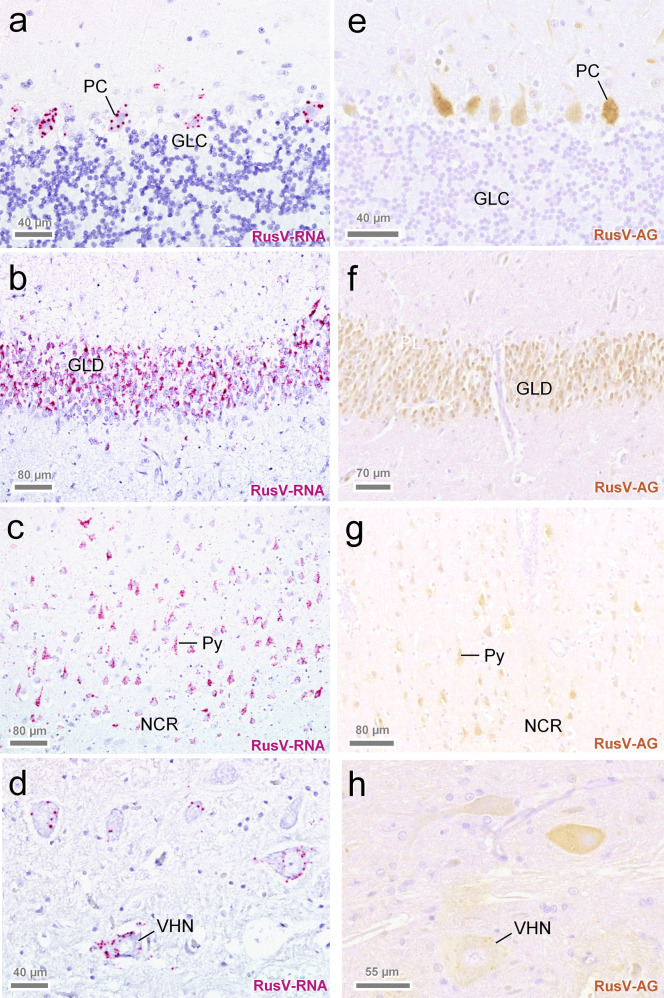
Detection of rustrela virus (RusV) RNA and antigen in the central nervous system of encephalitic cats. RusV RNA was detect by RNAscope in situ hybridization (**a**–**d**), while RusV capsid protein was detected by immunohistochemistry (**e**–**h**). Detection of rustrela virus (RusV) RNA by RNAscope in situ hybridization (**a**–**d**) and RusV antigen by immunohistochemistry (**e**–**h**) in the central nervous system of encephalitic cats. Both virus RNA and capsid protein were located mainly in the cytoplasm of different nerve cell populations. Typical are spherical reaction products, which may coalesce to more extensive and/or diffuse signal. Neurons with the highest viral load were particularly Purkinje cells (**a**, **e**: PC), granule cells of dentate gyrus (**b**, **f**: GLD), pyramidal cells across cerebral cortices including neocortex (**c**, **g**: Py). Also, numerous RusV-positive cells are seen in lower brain stem and spinal ventral horn neurons (**d**, **h**: VHN). GLC: granule cell layer of cerebellar cortex; GLD: granule cell layer of dentate gyrus; NCR: neocortical ribbon; PC: Purkinje cell; Py: pyramidal cell; VHN: ventral horn neuron. Cats: **a**, **e** SWE_03; **b**, **f** SWE_11; **c**, **g** SWE_05; **d**, **h** AUT_09. Representative images of RusV-infected cats are presented. All case and control cats (*n* = 29 each) were analysed. Results of IHC and ISH analyses are presented in Table 1 and Fig. 2.

To confirm and further characterize RusV infection in the cats, we employed viral RNA detection by ISH and antigen detection by IHC (Fig. 3). An RNAscope ISH probe was designed to target the highly conserved stretch at the 5’ terminus of the RusV genome (Supplementary Fig. 3). Specific ISH signal was observed in 24 out of 29 tested cats from all three countries (Table 1; Fig. 3a–d). Two animals revealed inconclusive results and two were ISH-negative (Fig. 2).

In addition, we performed IHC using a newly generated mouse monoclonal antibody targeting the RusV capsid protein. Specific immunostaining mirroring the ISH pattern (Fig. 3e–h) was seen in 27 out of 29 analysed cats with ‘staggering disease’, but not in any brain from 18 tested control cats (Table 1). IHC identified RusV antigen in two cases that had been negative by RT-qPCR from FFPE brain tissue (AUT_03 and GER_02), whereas one RT-qPCR-positive individual (AUT_05) remained negative by IHC (Fig. 2).

By both, ISH and IHC, a specific diffuse to granular intracellular chromogen signal was observed predominantly in perikarya of pyramidal neurons of cerebral cortices, namely of neocortex, cingulate/parahippocampal cortex (Fig. 3c, g), and hippocampus proper (Fig. 3f), in cell bodies of granule cells of dentate gyrus (Fig. 3b), Purkinje cells of the cerebellum (Fig. 3a, e), multipolar neurons of brain stem and cerebellar roof, and in ventral horn neurons of the spinal cord (Fig. 3d, h). On occasion, cytoplasmic immunoreactivity was also noted in individual interposed neuroglial and microglial cells. In addition, some small dot-like reactions were spotted in a scattered pattern amongst the neuropil and white matter.

To confirm the specificity of these findings, brain tissue from all 29 control cats was analysed by IHC and brains from 17 control cats representing all three countries were analysed by ISH. Neither viral antigen nor RusV RNA were detected in these animals (Table 1).

### Demographic data, clinical disease, and histopathology of RusV-infected cats

Among the 29 cats in this study that met the criteria of ‘staggering disease’, 27 cats were identified as RusV-positive by at least two of the employed methods (Table 1; Fig. 2). Twenty-one (77.8%) of them were neutered or intact males (Supplementary Table 2), which is consistent with previous studies on ‘staggering disease’^17,18,42^. All affected animals were adults, with a median age of 3.2 years (range 1.5 to 12.3; Supplementary Fig. 5a; Supplementary Table 2), and all had outdoor access (when reported) (Supplementary Table 2). The onset of disease had occurred more often in winter and spring (December to May: 18 cases) as compared to summer and fall (June to November: 7 cases; Supplementary Fig. 5b; Supplementary Table 2).

Typically observed clinical signs included gradually deteriorating gait abnormalities, with abnormal posture, stiff gait, ataxia, hind limb-predominant weakness, progressing to non-ambulatory tetraparesis and proprioceptive deficits. In addition, fever, behavioural changes such as abnormal vocalization or affectionate behaviour, depression, hyperaesthesia in dorsal back and lumbar/tail region, reduced spinal reflexes and postural reactions, signs of cranial nerve dysfunction and inability to retract claws were reported in some of the cases. In one animal, generalized seizures were specifically recorded (Supplementary Table 3). Duration from the reported disease onset to euthanasia ranged from two days to more than one year, with most of the cats being euthanized within less than two months (median two weeks) (Supplementary Fig. 5c, d; Supplementary Table 2).

**Fig. 4:**
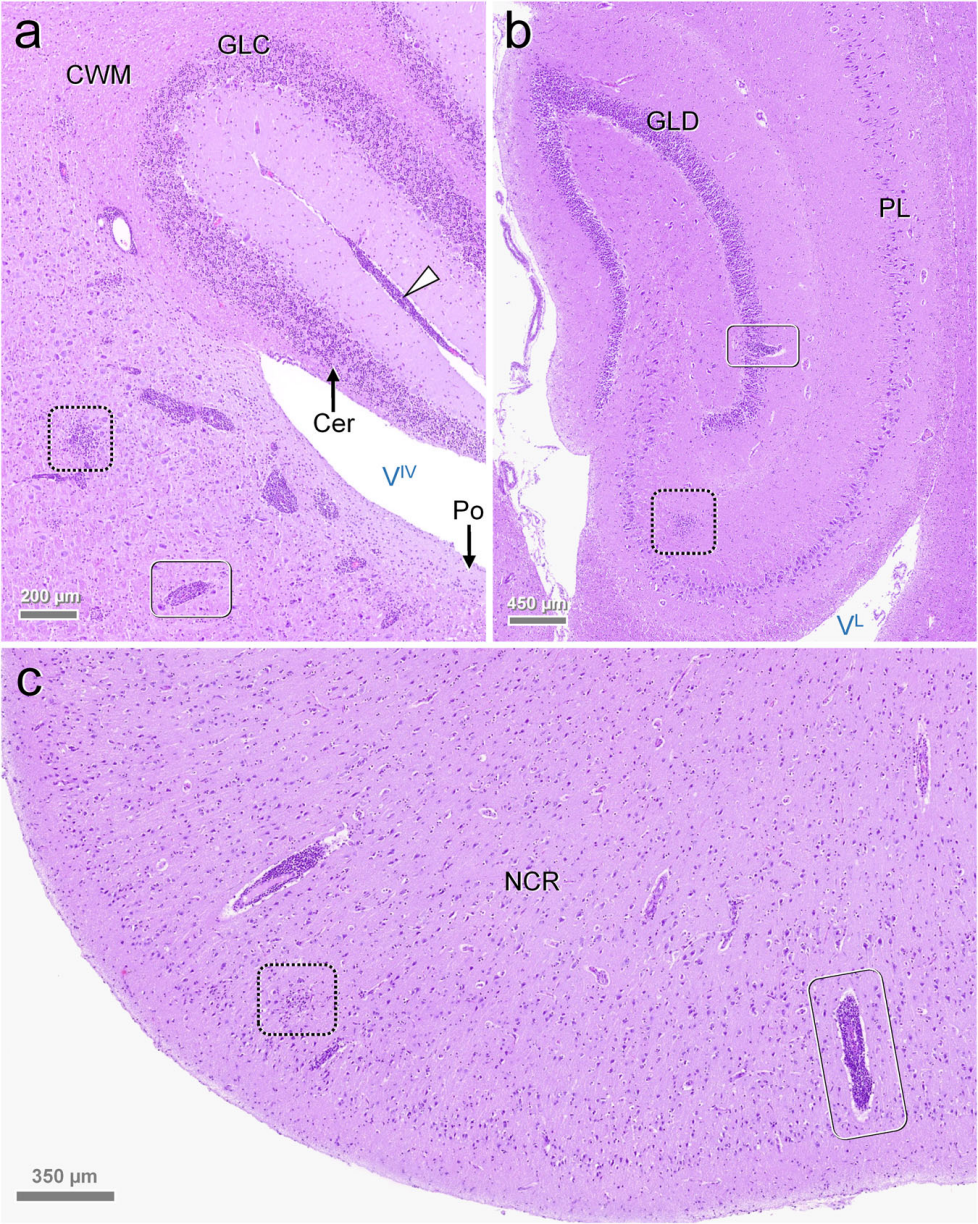
Encephalitic pattern in rustrela virus (RusV)-infected cats. Histology typically features polio-predominant, perivascular lymphohistiocytic cuffs (**a**–**c**: solid boxes) and angiocentric infiltrates (**a**–**c**: dashed boxes). They are most prominent in brain stem (**a**: Po), hippocampus formation (**b**) and neocortex (**c**). Leptomeningeal infiltrates (**a**: white arrowhead) also occur in areas with sparse parenchymal infiltration such as the cerebellum (**a**: Cer). Stain: haematoxylin eosin (H.E.). Anatomical landmarks: Cer: cerebellum; CWM: cerebellar white matter; GLC: granule cell layer of cerebellar cortex; GLD: granule cell layer of dentate gyrus; NCR: neocortical ribbon; PL: pyramidal cell layer; Po: pons; V^IV^: fourth ventricle; V^L^: lateral ventricle. Cats: **a** SWE_04; **b**, **c** SWE_07. Representative images of RusV-infected cats are presented. All case and control cats (*n* = 29 each) were analysed. Histopathological diagnoses are provided in Supplementary Tables 3 and 4.

**Fig. 5:**
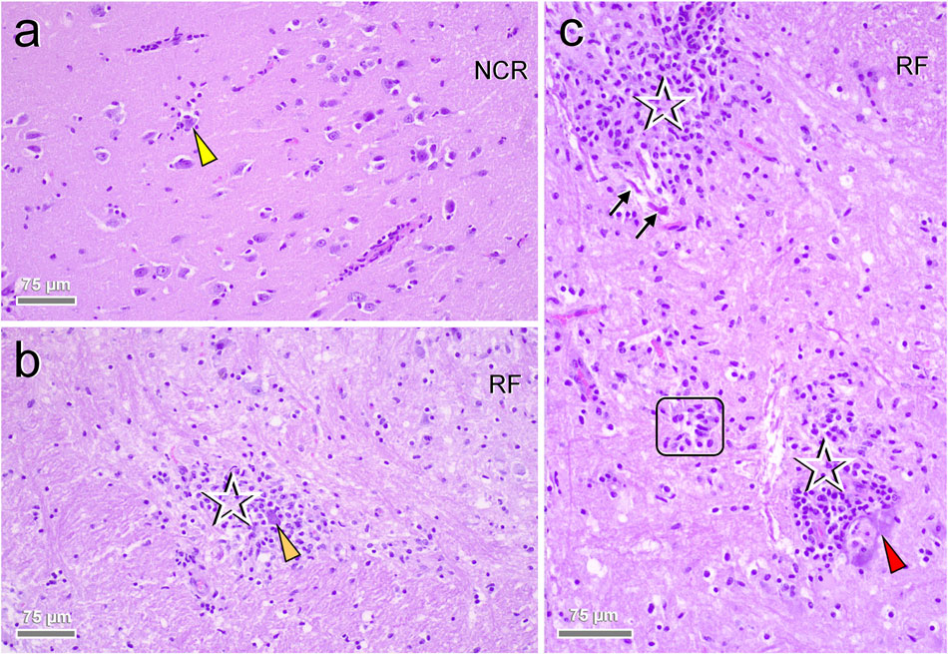
Close-up pathology and cellular damage of rustrela virus (RusV) infection within the brain. Infected brains show neurons (**a**–**c**: arrowheads) with (**c**: red arrowhead) and without (**a**, **b**: yellow and orange arrowheads) degenerative features, early (**a**, **b**: yellow and orange arrowheads) and advanced (**c**: red arrowhead) neuronophagia suggestive of a neuronotropic pathogen. Focal dropout of neurons is accompanied by microglial stars (**c**: frame). Inflammatory infiltrates (**b**, **c**: asterisks) mingle with focal glial proliferates. Dystrophic axons (**c**: black arrows) are occasionally present within the perilesional area. Stain: haematoxylin eosin (H.E.). Anatomical landmarks: NCR: neocortical ribbon; RF: reticular formation. Cats: **a** SWE_06; **b**, **c** SWE_04. Representative images of RusV-infected cats are presented. All case and control cats (*n* = 29 each) were analysed. Histopathological diagnoses are provided in Supplementary Tables 3 and 4.

In congruence with previous reports on feline ‘staggering disease’^11,12,18^, histologic examination of brain and spinal cord revealed widespread, polio-predominant angiocentric lymphocytic and/or lymphohistiocytic infiltrates throughout the cases (Figs. 4 and 5; Supplementary Table 3). Occasionally, they were accompanied by oligofocal astrogliosis and microglial proliferates, a few degenerating neurons and neuronophagic nodules (Fig. 5). Parenchymal inflammation was most pronounced in the brain stem (Figs. 4a, 5b, c), cerebral cortices (Fig. 4b, c), and all levels of the spinal cord, while they were less evident in the cerebellum despite often prominent detection of viral RNA and antigen (Fig. 4a). The predominance of inflammation in the grey matter was confirmed by Luxol Fast Blue-Cresyl Echt Violet stain for selected RusV-infected cats (Supplementary Fig. 6). In addition, lymphohistiocytic infiltrates and fewer plasma cells were present also in the leptomeninges, including those of the cerebellum (Fig. 4a). Notably, viral RNA and antigen signals were seen beyond areas affected by inflammatory changes (Fig. 3f) and occasionally inflammatory changes were also present in areas with viral RNA and antigen signals in the immediate vicinity. Throughout all regions, there were no inclusion bodies observed.

### Detection of RusV RNA in rodents from Southern Sweden

**Fig. 6:**
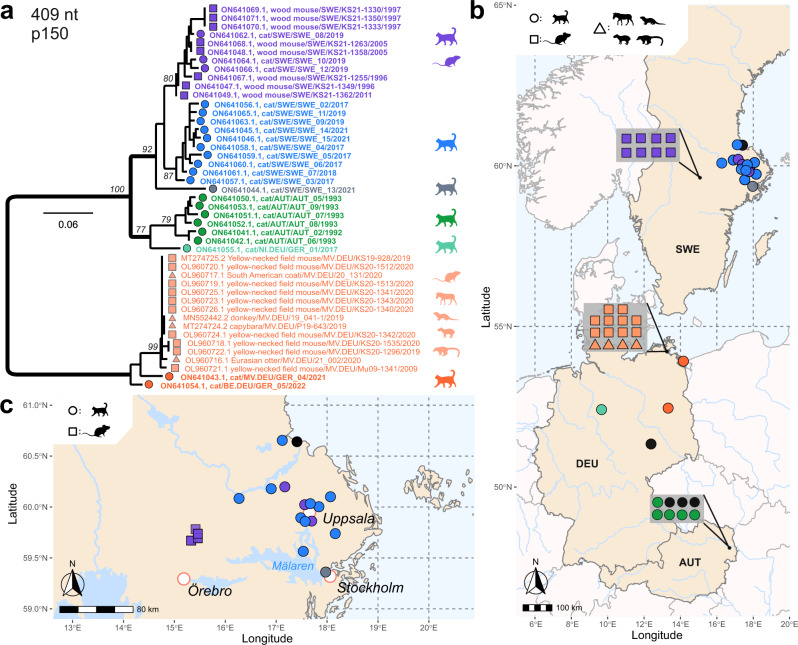
Phylogenetic analysis and spatial distribution of rustrela virus (RusV) infections in Europe. **a** Maximum-likelihood (ML) phylogenetic tree of partial RusV sequences (409 nucleotides, representing genome positions 100 to 508 of donkey-derived RusV reference genome MN552442.2; IQ-TREE version 2.2.0; TN + F + G4; 100,000 ultrafast bootstraps). Only bootstrap values ≥70 at major branches are shown in the phylogenetic tree. RusV sequence names are shown in the format “host/ISO 1366 code of location (federal state.country)/animal ID/year”. **b**, **c** Mapping of the geographic origin of RusV-positive animals in Europe (**b**) and in the Lake Mälaren region in Sweden (**c**). Colours represent the phylogenetic clades of the sequences (**a**). RusV-positive cats that failed to deliver sequences are depicted in black. The respective host animals are shown as circles (cats), squares (*Apodemus* spp.), and triangles (zoo animals). Symbols in grey boxes represent individuals from the same or very close locations. AUT Austria, DEU/GER Germany, SWE Sweden, BE Berlin, MV Mecklenburg-Western Pomerania, NI Lower Saxony.

We furthermore screened brain samples from 116 rodents that had been collected between 1995 and 2019 during monitoring studies near Grimsö in Örebro county (Supplementary Fig. 6), which is situated approximately 80 km Southwest of the origin of the closest RusV-infected cat detected in this study (Fig. 6). PanRusV RT-qPCR detected RusV RNA in eight out of 106 (7.5%) wood mice (synonym ‘long-tailed field mice’; *Apodemus sylvaticus*) with Cq values ranging from 20 to 35 (Supplementary Fig. 4b). In contrast, we did not detect RusV RNA in ten yellow-necked field mice from the same location. The positive individuals were collected in the years 1996 (*n* = 2), 1997 (*n* = 3), 2005 (*n* = 2) and 2011 (*n* = 1). All positive animals had been trapped during fall season, which is consistent with the considerably higher number of wood mice trapped during fall (*n* = 94) as compared to spring (*n* = 12; Supplementary Fig. 7).

None of the positive animals showed inflammatory lesions in their brain tissues. Sample quality allowed for ISH analysis of brain tissue for only four RusV-positive individuals. All of them exhibited specific signal, whereas one RT-qPCR-negative wood mouse did not when tested in parallel (Supplementary Fig. 8).

### Phylogenetic analysis and spatial distribution of RusV sequences from cats and wood mice

To allow for a detailed phylogenetic analysis, we aimed at generating RusV sequence information for all positive cats and wood mice. However, whole RusV genome sequencing by HTS is sophisticated and laborious^40^. Particularly for those individuals with only FFPE material available, the generated sequences were highly fragmented. Thus, we designed primers specifically targeting a stretch of 409 nt within the highly conserved region at the 5’ end of the genome to be applied for conventional RT-PCR and subsequent Sanger sequencing (Supplementary Table 1; Supplementary Fig. 3). Using this approach, sufficient sequence information was generated for 23 RusV-positive cats and all eight RusV-positive wood mice. Phylogenetic analysis of these sequences together with all previously published RusV sequences revealed three clearly distinguishable clades for sequences originating from Sweden, Austria, or northeastern Germany, with the Swedish and Austrian clades being more closely related to each other than to the northeastern German clade (Fig. 6a). The Swedish clade revealed three distinguishable subclades. One subclade harboured sequences from ten cats from an area of about 9000 km² around the city Uppsala. A second subclade included three RusV sequences from cats from the same region and all sequences from wood mice from Grimsö. The third subclade was constituted by only a single sequence from a cat from Stockholm (Fig. 6a, c). The sequences of both cats from northeastern Germany belonged to the previously published northeastern German clade (Fig. 6a, b)^37,40^. Surprisingly, sequence fragments available for cat GER_01, which originated from Hannover in Central Germany, were more closely related to sequences of the Austrian clade than to the northeastern German clade (Fig. 6a, b). This cat had been imported from China about one year before the onset of the disease, but it had never been to Austria^6^.

## Discussion

For almost five decades, ‘staggering disease’ in domestic cats had been suspected as a cohesive entity resulting in rather consistent clinical signs and histopathological patterns indicating a uniform, presumably viral, but still unknown aetiology^10–12,17,18^. While BoDV-1 had been discussed as a candidate for causing feline ‘staggering disease’^19,24,26–28^, proof of natural infections complying with current diagnostic standards could not be presented^20,31,32^. Here we used robust diagnostic approaches that had been demonstrated to successfully detect a broad range of orthobornaviruses^43^, including cases of BoDV-1-induced encephalitis in humans and domestic mammals^21,22,34^. Nevertheless, we were not able to detect bornavirus RNA or BoDV-1 nucleoprotein in any of the 29 tested cats from three different countries with clinicopathological features consistent with ‘staggering disease’. Thus, our results refute the hypothesis of BoDV-1 being the causative agent of ‘staggering disease’.

Instead, we were able to unequivocally confirm RusV infection in 27 out of these 29 cats. We consistently detected RusV RNA and antigen by employing independent diagnostic assays, including RT-qPCR, genome sequencing, ISH and IHC in the majority of these individuals. Only minor inconsistencies between results of the assays occurred, possibly due to genetic variability of the involved RusV variants, quality of the available material and differential sensitivities of the employed assays that may have led to false negative results of single tests, particularly for individuals for which only archived FFPE material was available. Experimental RusV infection of cats, to reproduce the disease and thereby fulfil Henle-Koch´s postulates, has not been performed so far due to the lack of a virus isolate. However, we demonstrate a striking association between infection and disease, with almost all animals of the ‘staggering disease’ group being RusV-positive, whereas the virus was not detected in any control cat without neurologic disease or with other types of encephalitis. Furthermore, clinical course and histologic lesions observed for cats classified to have ‘staggering disease’ in this and previous studies^10,12,17,18^ closely resembled those described for other RusV-infected mammals in Germany^37,40^. Thus, RusV should be considered as the causative agent of ‘staggering disease’, confirming the previous assumption that feline ‘staggering disease’ is a cohesive and unicausal disease entity rather than a heterogenic syndrome^11,18^. Provided that future studies further confirm our findings, we suggest that the criteria for the diagnosis of ‘staggering disease’, which is so far based on the typical histologic findings in combination with a consistent clinical presentation^11,18^, should be amended by the detection of a RusV infection. Since neither the clinical signs nor the histopathologic lesions of ‘staggering disease’ are pathognomonic, disorders resembling ‘staggering disease’ may be found to be unrelated to RusV, as demonstrated for animal GER_03 in this study. BoDV-1 is a potential causative agent of non-suppurative encephalitis of domestic cats in regions where this virus is endemic (parts of Germany, Austria, Switzerland and the Principality of Liechtenstein), but it has so far not been detected in Sweden or in the area of Vienna in Eastern Austria^20–22,31,32,44^. Furthermore, inflammatory lesions in the CNS of experimentally BoDV-1-infected cats were described to predominate in the white matter and do thus differ from the description of ‘staggering disease’^29^.

In congruence with observations from RusV-infected zoo animals^37,40^, RusV RNA and antigen in infected cats were detected predominantly in neurons of cerebral cortex, hippocampus, cerebellum, brain stem and spinal cord. Notably, the infection also frequently involved cerebellar cortex and cerebellar roof nuclei that essentially contribute to extrapyramidal coordination of gait and movement patterns^45^. Histologic lesions were likewise most prominent in the grey matter, in particular that of brain stem, cerebral cortex and spinal cord, which is consistent with previous findings for cats with ‘staggering disease’^12,18^ as well as RusV-infected mammals^37,40^. Neuropathogenic changes included neuronal degeneration, perineuronal astrogliosis, microglial proliferates and neuronophagia, but also evoked focal and distant inflammatory changes characterized predominantly by angiocentric lymphocytic and/or lymphohistiocytic infiltrates. As observed for other viral encephalitides, such as rabies or Borna disease, the most prominent inflammatory lesions were found not always in areas with highest number of RusV RNA- and antigen-positive cells^46,47^. Upon cell and tissue destruction, convection of antigens via the glymphatic system in particular is assumed to trigger perivascular cuffs along the extracellular drainage route of Robin-Virchow spaces and the subarachnoid compartment^48^. Moreover, viral RNA and antigen may even be barely detectable in the brain parenchyma at the time of investigation due to virus clearance, as documented in flavivirus-associated encephalomyelitis^49,50^.

We detected RusV infection of cats in the Lake Mälaren region in Sweden and Northeast of Vienna in Austria, two traditional hotspots of ‘staggering disease’^10–12,18,42^, as well as in northern Germany, where RusV had been initially discovered^37,40^, but ‘staggering disease’ had not yet been reported. Phylogenetic analyses revealed the RusV sequences from the three regions to belong to separate genetic clusters, with the Swedish and Austrian sequences being more closely related to each other than to those from northern Germany. The considerable genetic variability with down to 75% nt sequence identity among the different lineages posed a major challenge for the generation of broadly reactive diagnostic tools. However, a particularly conserved sequence stretch at the 5’ terminus of the genome allowed for the design and application of versatile primers and probes for PCR and ISH assays. Furthermore, a monoclonal mouse antibody targeting the RusV capsid protein proved suitable for the detection of all three major RusV lineages.

While yellow-necked field mice are considered as putative reservoir hosts of RusV in northern Germany^37,40^, we surprisingly detected RusV in Sweden only in the closely related wood mice but not in yellow-necked field mice from the same area in Örebro county. Since the majority of tested individuals from this location were wood mice, it remains to be elucidated whether this discrepancy is mainly a result of different species compositions of the analysed sample collections or whether it represents a diverging adaptation of RusV variants to alternative rodent reservoir hosts.

The route of RusV transmission within its presumed reservoir as well as from there to other hosts remains unknown. The tissue tropism in zoo animals and yellow-necked field mice in Germany was described as restricted almost exclusively to the CNS, with occasional detection of RusV RNA in peripheral nerve fibres. Viral shedding has not been described so far^37,40^. In the future, detailed data on tissue distribution needs to be obtained also for RusV-infected cats and wood mice, but this was beyond the scope of this study. Furthermore, the possibility of RusV shedding by infected cats remains to be elucidated. However, the apparently spatially restricted occurrence of the phylogenetic clusters argues in favour of a continuous viral spread only within a locally bound reservoir, such as small mammals, whereas more mobile hosts, including domestic animals that may be transported over long distances, serve predominantly as erroneous dead-end hosts. Similar patterns have been evidenced for shrew and rodent reservoir-bound viruses such as BoDV-1, rat hepatitis E virus or Puumala orthohantavirus^32,44,51,52^. The sporadic occurrence of ‘staggering disease’ in domestic cat populations, the apparent lack of outbreak series within cat holdings, as well as the almost exclusive restriction to cats with outdoor access, often originating from rural areas, further support this assumption^12,17,18,42^.

Previous studies suggested a seasonal occurrence of ‘staggering disease’ with more cases in winter and spring than summer and fall^18^. Although higher case numbers and a more systematic sampling scheme are required for solid statistical evaluation, the same tendency was observed also in our study. This seasonal pattern may be attributable to fluctuating reservoir populations. During the small mammal monitoring in Grimsö, Sweden, numbers of *Apodemus* spp. trapped in fall were much higher than in spring. In addition, movement of small rodents towards and into human dwellings during the winter is frequently reported and has been discussed to be associated with transmission of zoonotic pathogens such as Puumala orthohantavirus to humans^53^. Increased exposure to *Apodemus* spp. during the fall and winter season might also facilitate RusV transmission to cats. However, since the incubation period of RusV-induced disease is unknown, time points of infection cannot be reliably estimated so far. Changes of reservoir populations may also explain long-term temporal patterns of ‘staggering disease’ occurrence. While cases have been continuously observed in the Swedish Lake Mälaren region from at least the 1970s until today^10,18,27,28,42^, ‘staggering disease’ in the districts north-east of Vienna was diagnosed mainly during the early 1990s^11,12^ and appears to have ceased thereafter.

In summary, we provide convincing evidence of association of RusV infection with ‘staggering disease’ in cats, supporting a causative role. Our results demonstrate a much broader genetic diversity and spatial distribution of RusV than initially appreciated, and we identified the wood mouse as an additional potential reservoir host. The availability of broadly reactive diagnostic tools may lead to the detection of RusV in encephalitic cats also in regions where ‘staggering disease’ has not been evident before. Furthermore, given the broad range of affected zoo animals^37,40^, RusV may be responsible also for additional neurologic disorders in other mammalian species, possibly even humans. In addition to establishing further diagnostic tools, including serological assays, further studies on its epidemiology and attempts to isolate the virus and establish infection models, future research should include the evaluation of a possible zoonotic potential of RusV.

## Methods

### Samples and data collection

Fresh-frozen or FFPE brain and/or spinal cord samples from 29 cats fulfilling the inclusion criteria for this study (multifocal lymphohistiocytic meningoencephalomyelitis or meningoencephalitis—if spinal cord was not available for analysis—with predominance in the grey matter of unknown cause, in combination with clinical signs compatible with the description of ‘staggering disease’, particularly ataxia and other gait disturbances) were provided by different laboratories from Sweden, Austria, and Germany (Table 1; Fig. 2; Supplementary Tables 2 and 3). Exclusion criteria were inflammatory changes predominantly affecting the white matter as well as extensive degenerative grey and white matter lesions. The samples dated back to 1991 to 1993 (Austria) or 2017 to 2022 (Germany and Sweden). Some of these cases had been published previously^6,11,12^. In addition, frozen brain samples from 21 cats originating from Sweden, Austria, and Germany without encephalitis were included as controls. An additional control group was composed of frozen or FFPE brain samples from eight cats from Germany that had suffered from encephalitis of other types or causes, such as CNS manifestation of feline coronavirus (FCoV)-associated feline infectious peritonitis (FIP), vasculitic disorders of suspected hypertensive background and immune-mediated limbic encephalitis (Table 1; Supplementary Table 4)^6^. Metadata were provided by the submitters and/or extracted from the previous publications, including course and duration of disease, age, sex, origin, and outdoor access of the cats (Supplementary Tables 2 and 4), as well as clinical signs (Supplementary Table 3).

Furthermore, the study includes archived frozen brain samples from yellow-necked field mice (*A. flavicollis*; *n* = 10) and wood mice (*A. sylvaticus*; *n* = 106) that had been collected near Grimsö, Örebro county, Sweden, as part of the Swedish Environmental Monitoring Program of Small Rodents and in cooperation with the Swedish Infrastructure for Ecosystem Science (SITES) at Grimsö Wildlife Research Station^54^. Trapping was approved by the Swedish Environmental Protection Agency (latest permission: NV-412-4009-10) and the Animal Ethics Committee in Umeå (latest permissions: Dnr A 61–11), and all applicable institutional and national guidelines for the use of animals were followed. Species identities were confirmed by cytochrome *b* gene sequencing as described previously^55^.

### RNA extraction

Fresh-frozen samples were mechanically disrupted in 1 ml TRIzol Reagent (Life Technologies, Darmstadt, Germany) by using the TissueLyser II (Qiagen, Hilden, Germany) according to the manufacturers’ instructions. After the addition of 200 µl chloroform and a centrifugation step (14,000 × *g*, 10 min, 4 °C), the aqueous phase was collected and added to 250 µl isopropanol. Total RNA was extracted using the silica bead-based NucleoMagVet kit (Macherey & Nagel, Düren, Germany) with the KingFisher™ Flex Purification System (Thermo Fisher Scientific, Waltham, MA, USA) according to the manufacturers’ instructions. In vitro-transcribed RNA of the enhanced green fluorescent protein (eGFP) gene was added during the extraction procedure as described by Hoffmann et al.^56^.

RNA extraction from FFPE tissue was performed with a combination of truXTRAC FFPE total NA Kit (Covaris, Brighton, UK) and the Agencourt RNAdvance Tissue Kit (Beckman Coulter, Krefeld, Germany). FFPE sections of 3 µm thickness were loaded into a microTUBE-130 Screw-Cap (Covaris) together with 110 µl Tissue Lysis Buffer and 10 µl proteinase K solution (both Covaris). The lysate was processed with a M220 Focused ultrasonicator (Covaris) according to the manufacturer’s recommendations for acoustic pellet resuspension. The tube was subsequently incubated at 56 °C in a thermal shaker at 300 rpm overnight (no longer than 18 h). Subsequently, the sample tube was cooled to room temperature and centrifuged at 5000 × *g* for 15 min using a microTUBE-130 centrifuge adapter. A volume of 100 µl supernatant was transferred into a clean 1.5 ml reaction tube without transferring any wax or paraffin. After another centrifugation (5 min at 20,000 × *g*), 85 µl of the lower phase with the RNA-containing tissue pellet was transferred into a clean 1.5 ml reaction tube. It was incubated at 80 °C for 20 min and then cooled to room temperature, before 175 µl B1 buffer (Covaris) were added, mixed, and briefly centrifuged. Thereafter, 250 µl of 65% isopropanol were added, mixed, and briefly centrifuged. Subsequently, the preparations were further processed with the Agencourt RNAdvance Tissue Kit (Beckman Coulter) with the KingFisher™ Flex Purification System (Thermo Fisher Scientific) according to the manufacturer’s instructions.

### Metagenomic analysis and complete genome sequencing by HTS

Total RNA was sequenced using a universal metagenomics sequencing workflow^41^. In brief, total RNA was extracted from fresh-frozen tissue samples using a cryoPREP impactor (Covaris) along with the Agencourt RNAdvance Tissue Kit (Beckman Coulter) and a KingFisher Flex Purification System (Thermo Fisher Scientific). Then, 350 ng RNA per sample were reverse-transcribed into cDNA using the SuperScript IV First-Strand cDNA Synthesis System (Thermo Fisher Scientific) and the NEBNext Ultra II Non-Directional RNA Second Strand Synthesis Module (New England Biolabs, Ipswich, MA, USA). Subsequently, cDNA was processed to generate barcoded sequencing libraries as described in detail elsewhere^41^. The cDNA was fragmented to 200 base pairs (bp) length (for FFPE material) or 500 bp length (for fresh-frozen material) using an M220 Focused ultrasonicator (Covaris). Subsequent library preparation was performed as described previously, with the following modification for FFPE material during size exclusion: small fragments were retained and purified twice with 1.2× Ampure XP Beads (Beckman Coulter). Libraries were quantified with the QIAseq Library Quant Assay Kit (Qiagen) and sequenced on an Ion Torrent S5XL instrument using Ion 530 chips and chemistry for 400 bp reads, or Ion 540 chips and chemistry for 200 bp reads (Thermo Fisher Scientific) for fresh-frozen or FFPE material, respectively. In addition to the original sequencing libraries, 7 µl of the libraries were used to apply a capture enrichment with the panRubi v2 myBaits panel as described elsewhere^40^. For samples with expected major sequence divergence (>20%) from the initially available RusV sequences from northeastern Germany that were used for designing the panRubi v2 myBaits panel, a hybridization temperature of 61 °C was used for 24–26 h. In addition, a new panRubi myBaits panel was designed (v3) adding preliminary genome information from samples of Sweden and Austria to the v2 panel. The panRubi v3 myBaits panel consists of 19,982 baits (60-nt oligonucleotides arranged every 20 nt, 3× tiling; GC content of 67.3%) and was collapsed at 98% sequence identity. This panel was applied with a hybridization temperature of 64 °C.

For selected RusV-positive samples, we additionally applied a depletion protocol in order to decrease the amount of host-derived ribosomal RNA (rRNA) within the total RNA and thereby increase the virus-to-background ratio. In detail, we used a pan-Mammal riboPOOL reaction kit (siTOOLs Biotech, Planegg, Germany) for 0.2 and 1 µg total RNA following the manufacturer´s instructions. The rRNA-depleted RNA was then used for strand-specific library preparation with the Collibri Stranded RNA Library Prep Kit (Thermo Fisher Scientific). The libraries were checked for sufficient quality and quantity using the 4150 TapeStation System (Agilent Technologies, Santa Clara, CA, USA) with the High Sensitivity D1000 ScreenTape and reagents (Agilent Technologies) as well as a Qubit Fluorometer (Thermo Fisher Scientific) along with the dsDNA HS Assay Kit (Thermo Fisher Scientific). Pooled libraries were sequenced using a NovaSeq 6000 (Illumina, San Diego, CA, USA) running in 100 bp mode.

### De novo assembly and sequence annotation of HTS-derived sequences

The raw sequences from Ion Torrent and Illumina systems were processed as described previously^40^. Briefly, the platform-specific adapters were initially removed from the reads and the sequences were trimmed according to their quality using either 454 Sequencing Systems Software (version 3.0) or Trim Galore (version 0.6.6)^57^ with automated adapter selection, for Ion Torrent and Illumina reads, respectively. Subsequently, the reads were filtered according to their average G + C content using PRINSEQ-lite (version 0.20.4)^58^ with a G + C threshold of ≥60 mol%. The trimmed and filtered reads were used for de novo assembly using SPAdes genome assembler (version 3.15.2)^59^ running in single cell mode (--sc) and Ion Torrent mode (--iontorrent) as required. Subsequently, all contigs were mapped back to an appropriate RusV reference sequence using Geneious generic mapper with medium sensitivity (Geneious Prime 2021.0.1; Biomatters, Auckland, New Zealand), and a consensus sequence was generated. The final sequence was annotated according to an appropriate RusV reference genome using ORF detection as provided by Geneious Prime 2021.0.1.

### Bornavirus and RusV RT-qPCRs and design of adapted broad range RusV-specific primers and probes

Two RT-qPCR assays were applied for the detection of either a broad range of orthobornaviruses (panBorna v7.2; Supplementary Table 1) or specifically BoDV-1 (BoDV-1 Mix-1; Supplementary Table 1) following previously published procedures^21,34^. Initial screening for RusV-specific RNA was performed using a TaqMan-based RT-qPCR assay (RusV Mix-1; Supplementary Table 1) targeting the initially discovered RusV sequences from a zoo in northeastern Germany as described by Bennett et al.^37^. The exogenously supplemented eGFP RNA was amplified as RNA extraction control as described previously^56^.

To establish a new RT-qPCR assay for the detection of a broader range of RusV sequences, all available sequences from northeastern Germany and Sweden were aligned and a set of primers and probe (panRusV-2; Supplementary Table 1; Supplementary Fig. 3) was designed to target a highly conserved region at the 5’ terminus of the genome. RT-qPCR was performed with AgPath-ID One-Step RT-PCR reagents (Thermo Fisher Scientific), panRusV-2 primers (final concentration: 0.8 µM each) and probe (0.4 µM), eGFP primers (0.2 µM each) and probe (0.15 µM), and 2.5 µl extracted RNA in a total volume of 12.5 µl. The reaction was performed with the following cycler setup: 45 °C for 10 min, 95 °C for 10 min, 45 cycles of 95 °C for 15 s, 60 °C for 30 s and 72 °C for 30 s. A standard RNA preparation of a RusV-positive donkey brain^37^ served as positive control and was used for the calibration of Cq values in each RT-qPCR analysis.

### Determination of partial p150-encoding RusV sequences by Sanger sequencing

Highly conserved primer binding sites in the same alignment as described above were also identified for the amplification of 449 nt at the 5’ end of the p150-encoding sequence by conventional RT-PCR (Supplementary Table 1; Supplementary Fig. 3). RNA extracted from frozen brain samples from all cats and rodents with positive panRusV RT-qPCR results was analysed using the following One-Step RT-PCR conditions: 2.5 μl RNA were amplified in a total volume of 25 μl using the SuperScript III One-Step RT-PCR system with Platinum Taq DNA polymerase (Thermo Fisher Scientific) and 0.4 µM each of primers RusV_80+ and RusV_528- (Supplementary Table 1; Supplementary Fig. 3). The cycler setup consisted of 50 °C for 30 min, 94 °C for 2 min, followed by 40 cycles of 94 °C for 30 s, 63 °C for 30 s, and 68 °C for 25 s, and a final elongation step at 68 °C for 5 min. Following separation and visualization by gel electrophoresis, amplification products were purified using Zymoclean Gel DNA Recovery Kit (Zymo Research, Freiburg, Germany) and Sanger sequencing service was provided by Microsynth Seqlab (Balgach, Switzerland). Amplicons were sequenced in both directions and consensus sequences of 409 bp lengths were generated after de novo assembly of quality- and primer-trimmed raw sequences in Geneious Prime 2021.0.1.

### Phylogeny and geographic mappings

Phylogenetic analysis of RusV sequences generated in this study was performed together with representative sequences of all currently known matonaviruses^37,60^, as well as all publicly available RusV sequences from the INSDC database^37,40^. For the phylogeny within the known matonaviruses, the aa sequences of the sPP were aligned using MUSCLE (version 3.8.425)^61^ with a maximum of 100 iterations. A maximum-likelihood (ML) phylogenetic tree was then calculated using IQ-TREE2 (version 2.2.0)^62^ running in automatic model selection mode (FLU + F + I + G4) and applying 100,000 ultrafast bootstrap replicates^63^. For phylogenetic analysis of RusV nt sequences, the complete or nearly complete RusV genome sequences were aligned using MAFFT (version 7.450)^64^. A ML tree was then calculated as described above (model: TIM3 + F + I). The alignment was further used for sequence comparison with a sliding window approach that calculated the pairwise distances (Jukes Cantor 1969 model) within a window of 200 nt every 50 nt. A phylogenetic tree of partial p150 protein-coding sequences of 409 nt length was built as described above (model: TN + F + G4).

### Histologic examination

Brain and spinal cord samples of cats were harvested on post mortem examination via extensive craniectomy-laminectomy. For histology, brain and spinal cord tissues from cats as well as brain tissue from all eight RusV-positive wood mice were fixed in 10% neutral-buffered formalin. Fixed neural tissues were routinely sampled, processed in an automatic tissue processor, embedded in paraffin wax, sectioned at 2–4 μm, and stained with histological standard stains including haematoxylin eosin (H.E.). A selection of brains from four staggering and seven control cats underwent Luxol Fast Blue-Cresyl Echt Violet stain as described by Klüver and Barrera^65^ (Supplementary Fig. 6).

Sections were microscopically examined for the presence of non-suppurative, lymphohistiocytic encephalitis, meningoencephalitis and/or meningoencephalomyelitis. Inflammation was graded mild to severe based on the extent of inflammatory cell infiltrates. Mild encephalitis comprised few perivascular infiltrates, most of which showed one to two layers of cells and were not necessarily present in all investigated locations. One or two larger infiltrates in a single location were allowed to occur in this category. Moderate encephalitis comprised several infiltrates per location, showing three to five layers of cells, allowing single locations with larger or smaller infiltrates. Severe encephalitis comprised many perivascular infiltrates, most of which showed several layers of cells (>5) in the majority of investigated locations.

### Detection of RusV-specific RNA by ISH

A custom-designed RNAscope probe was provided by Advanced Cell Diagnostics (Newark, NJ, USA) based on the consensus sequence of the available RusV sequences from Sweden, targeting the highly conserved region at the 5’ end of the RusV genome (catalogue no. 1145591-C1). A probe targeting the messenger RNA (mRNA) of the ubiquitous, widely expressed housekeeping gene peptidyl-prolyl-isomerase-B (*Felis catus*-PPIB; cat. no. 455011) was used as positive control, while a probe targeting bacterial dihydropicolinate reductase (DapB; cat. no. 310043) was used as a negative control probe. Viral nucleic acid was determined using ISH with the manual RNAscope 2.5 High Definition RED assay (Advanced Cell Diagnostics) according to the manufacturer’s instructions. Briefly, brain sections were deparaffinized and pre-treated with 1× Target Retrieval solution and RNAscope® Protease Plus solution prior to hybridization with the target probe. Subsequently, the tissue was treated with a series of pre-amplifiers and amplifiers followed by the application of a chromogenic substrate. The samples were counterstained with Hematoxylin Gill No. 2 (Merck, Darmstadt, Germany).

Brain sections of a RusV-positive capybara^37^ served as positive control and showed positive reactivity with the specific RusV RNAscope probe. A brain sample from a RusV-negative control cat incubated with the RusV RNAscope probe and a brain sample from a RusV-positive cat incubated with an irrelevant RNAscope probe (*Mycoplasma hyopneumoniae*) served as negative controls and yielded no reactivity. The scoring of the signals was performed as described in Table 2.

**Table 2:** Scoring of RNAscope ISH and IHC signal in brain slices

Score	Description
Negative	No specific staining detectable
Uncertain	Very few signals, not clearly associated with cellular structures
1	Signals in the cytoplasm or processes of singular neurons or glial cells
2	Signals in an average of 5–10 cells per field (in 40-fold magnification in a 0.237 mm^2^ field area)
3	Signals in an average of more than 10 cells (up to ~100) per field (in 40-fold magnification in a 0.237 mm^2^ field area)

### Recombinant protein production and generation of a monoclonal anti-RusV capsid protein antibody

A synthetic DNA string fragment encoding aa 128 to 308 of the RusV capsid protein, based on the sequence from an infected donkey from northeastern Germany (accession number MN552442.1), was ordered from GeneArt Gene synthesis (Thermo Fisher Scientific) and inserted into the pEXPR103 expression vector (IBA Lifesciences, Göttingen, Germany) in-frame with a Strep-tag-coding sequence at the 3′ end. The protein with a C-terminal Strep-tag was expressed in human Expi293 cells (Thermo Fisher Scientific) and subsequently purified using Strep-Tactin XT Superflow high capacity resin (IBA Lifesciences) following the manufacturer’s instructions.

For monoclonal antibody generation, two female BALB/c mice, aged four and twelve months, originating from the specific pathogen-free breeding unit of the Friedrich-Loeffler-Institut, were immunized intraperitoneally with 20 μg of purified capsid protein. The animals were kept in type II L cages at 12 h dark/light cycle, 20 to 24 °C room temperature and 45 to 65% humidity. The work was performed in compliance with the national and European legislation, with approval by the competent authority of the Federal State of Mecklenburg-Western Pomerania, Germany (reference number: 7221.3-2-042/17). The immunization, as well as the generation of hybridoma cells were performed as described previously^66^. The final RusV capsid-specific monoclonal antibody 2H11B1 was identified by indirect ELISA and immunofluorescence staining of transfected RK-13 rabbit kidney cells expressing the recombinant RusV capsid protein.

### Detection of RusV and BoDV-1 antigen by IHC

Brain sections were evaluated for expression of RusV capsid protein using the mouse monoclonal primary antibody 2H11B1. The slides were deparaffinised and underwent antigen retrieval in the microwave (750 W, 20 min) being immersed in 10 mM citrate buffer (pH 6.0) before incubation with the primary antibody (dilution 1:100) at 4 °C for 18 h. Successful labelling was demonstrated using ImmPRESS® polymer anti-mouse IgG (LINARIS Biologische Produkte, Dossenheim, Germany), coupled to peroxidase, and a diaminobenzidine tetrahydrochloride staining kit (ImmPACT DAB substrate HRP; BIOZOL Diagnostica, Eching, Germany) according to the manufacturers’ instructions. After peroxidase reaction, sections were counterstained with haematoxylin. Sections of a RusV-positive capybara brain^37^ served as virus-positive tissue control, whereas brain sections of cats from the control groups that had tested negative for RusV by RT-qPCR served as negative tissue control (Supplementary Fig. 8). Specificity of the anti-mouse IgG polymer was evaluated by two sections each of capybara brain and of RT-PCR-confirmed RusV-positive cat SWE_07, in which 2H11B1 antibody was replaced by horse serum and by anti-FCoV mouse monoclonal antibody (FIPV 3–70, LINARIS Biologische Produkte; Supplementary Fig. 8). The scoring of the signals was performed as described in Table 2.

Cat brain sections were furthermore assessed for the expression of BoDV-1 nucleoprotein using murine monoclonal antibody Bo18 (obtained from the Friedrich-Loeffler-Institut, Greifswald, Germany)^67^ with the ABC detection kit (biotinylated goat anti-mouse IgG; BIOZOL Diagnostica) and diaminobenzidine tetrahydrochloride (ImmPACT DAB substrate HRP; BIOZOL Diagnostica). Brain sections of a horse confirmed as BoDV-1-infected by RT-qPCR served as positive control (Supplementary Fig. 1). Replacement of Bo18 antibody by an irrelevant mouse monoclonal antibody (FIPV 3–70) was used as negative reagent control on BoDV-1-positive horse tissue and RusV-positive feline brain SWE_07.

## Supplementary information

Supplementary Information

## Data Availability

All RusV sequences generated during this study have been made publicly available in the INSDC database under the accession numbers ON641041 to ON641071. Accession numbers of additional sequences derived from public databases are provided in the text and/or in the phylogenetic tree (Fig. 6a). The data available on the animals included in this study as well as the diagnostic results generated during this study are provided in the Supplementary Information/Source data file. Source data are provided with this paper.

## Source data

Source Data
