# Supplementary material for: Mystery of fatal ‘staggering disease’ unravelled: novel rustrela virus causes severe meningoencephalomyelitis in domestic cats
Source: Nat Commun. 2023 Feb 4;14:624. doi: 10.1038/s41467-023-36204-w (PMC9899117; doi:10.1038/s41467-023-36204-w)
Supplement: Supplementary file 1 — Supplementary Information [file 41467_2023_36204_MOESM1_ESM.pdf]

Supplementary Information

Mystery of fatal 'Staggering disease' unravelled: Novel rustrela virus causes severe meningoencephalomyelitis in domestic cats

Authors:

Kaspar Matiasek, Florian Pfaff, Herbert Weissenböck, Claudia Wylezich, Jolanta Kolodziejek, Sofia Tengstrand, Frauke Ecke, Sina Nippert, Philip Starcky, Benedikt Litz, Jasmin Nessler, Peter Wohlsein, Christina Baumbach, Lars Mundhenk, Andrea Aebischer, Sven Reiche, Pia Weidinger, Karin M. Olofsson, Cecilia Rohdin, Christiane Weissenbacher-Lang, Julia Matt, Marco Rosati, Thomas Flegel, Birger Hörnfeldt, Dirk Höper, Rainer G. Ulrich, Norbert Nowotny, Martin Beer, Cecilia Ley, Dennis Rubbenstroth

Table of contents:

Supplementary Figures 1 to 8

Supplementary Tables 1 to 4

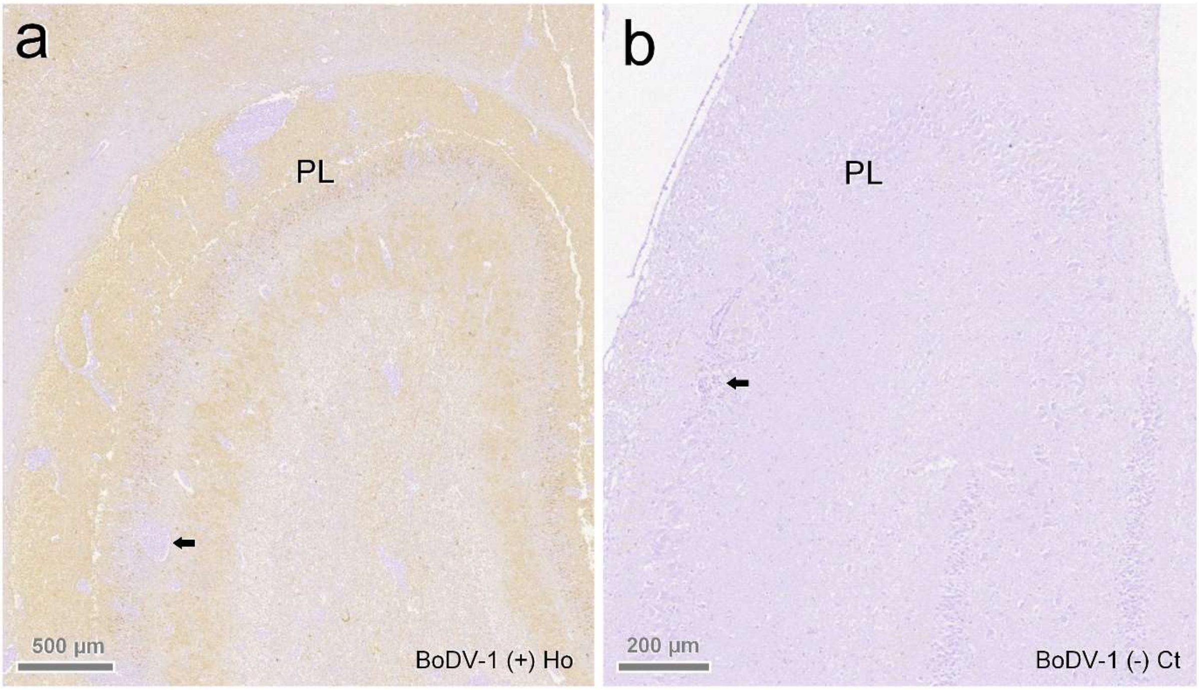

Supplementary Figure 1. Absence of Borna disease virus 1 (BoDV-1) antigen in brains of cats with 'staggering disease'. (**a**) Horses with Borna disease encephalitis show extensive immunopositivity for BoDV-1 nucleoprotein particularly in the hippocampus. (**b**) No BoDV-1 staining was seen in any of the tested cats, even with inflammatory infiltrates extending into hippocampus (arrow). Ho: horse; Ct: cat; PL: pyramidal cell layer of hippocampus; arrows: angiocentric infiltrates. Source: (**b**) cat SWE_01. A representative image of a RusV-infected cat is presented. All case and control cats (n=29 each) were analysed with negative results.

			1	2	3	4	5	6	7	8	9	10	11	12	13	14
			MN552442.2	MT274724.2	OL960721.1	OL960716.1	OL960722.1	**GER_04**	**AUT_02**	**AUT_06**	**SWE_13**	**SWE_14**	**SWE_15**	**KS21-1349**	**KS21-1362**	**KS21-1358**
**1**	MN552442.2	donkey/MV.DEU(19_041-1/2019		99.80	99.30	99.00	97.70	92.10	76.60	75.80	76.80	76.80	76.70	77.00	77.10	76.90
**2**	MT274724.2	Capybara/MV.DEU/P19-643/2019/Germany	99.80		99.20	98.90	97.70	92.10	76.50	75.70	76.80	76.70	76.70	77.00	77.00	76.80
**3**	OL960721.1	yellow-necked field mouse/MV.DEU/Mu09-1341/2009	99.30	99.20		99.00	97.70	92.10	76.60	75.90	76.80	76.80	76.80	77.00	77.10	76.90
**4**	OL960716.1	Eurasian otter/MV.DEU/21_002/2020	99.00	98.90	99.00		97.40	92.00	76.70	75.80	76.80	76.70	76.60	77.00	77.10	76.80
**5**	OL960722.1	yellow-necked field mouse/MV.DEU/KS20-1296/2020	97.70	97.70	97.70	97.40		92.20	76.50	75.70	76.80	76.80	76.70	76.90	76.90	76.70
**6**	**ON641043**	**cat/MV.DEU/GER_04/2021**	92.10	92.10	92.10	92.00	92.20		76.40	75.70	77.00	76.50	76.40	76.80	76.80	76.80
**7**	**ON641041**	**cat/AUT/AUT_02/1992**	76.60	76.50	76.60	76.70	76.50	76.40		97.50	82.40	81.60	81.70	82.00	82.10	82.10
**8**	**ON641042**	**cat/AUT/AUT_06/1993**	75.80	75.70	75.90	75.80	75.70	75.70	97.50		81.70	80.70	80.70	81.20	81.20	81.20
**9**	**ON641044**	**cat/SWE/SWE_13/2021**	76.80	76.80	76.80	76.80	76.80	77.00	82.40	81.70		85.90	85.90	86.10	86.00	86.10
**10**	**ON641045**	**cat/SWE/SWE_14/2021**	76.80	76.70	76.80	76.70	76.80	76.50	81.60	80.70	85.90		99.50	86.50	86.50	86.60
**11**	**ON641046**	**cat/SWE/SWE_15/2021**	76.70	76.70	76.80	76.60	76.70	76.40	81.70	80.70	85.90	99.50		86.70	86.60	86.70
**12**	**ON641047**	**wood mouse/SWE/KS21-1349/1996**	77.00	77.00	77.00	77.00	76.90	76.80	82.00	81.20	86.10	86.50	86.70		99.10	97.00
**13**	**ON641048**	**wood mouse/SWE/KS21-1362/2011**	77.10	77.00	77.10	77.10	76.90	76.80	82.10	81.20	86.00	86.50	86.60	99.10		96.70
**14**	**ON641049**	**wood mouse/SWE/KS21-1358/2005**	76.90	76.80	76.90	76.80	76.70	76.80	82.10	81.20	86.10	86.60	86.70	97.00	96.70	

Supplementary Figure 2. Nucleotide sequence identity matrix of an alignment of complete or nearly complete rustrela virus (RusV) genome sequences including five sequences established in this study. Sequences generated during this study are depicted in bold. Due to the high genetic similarity of the previously published sequences from Northeastern Germany[1, 2, 3], only five out of 14 available sequences are shown. RusV sequence names are shown in the format "host/ISO 1366 code of location (federal state.country)/animal ID/year".

AUT: Austria; DEU/GER: Germany; SWE: Sweden; MV: Mecklenburg-Western Pomerania.

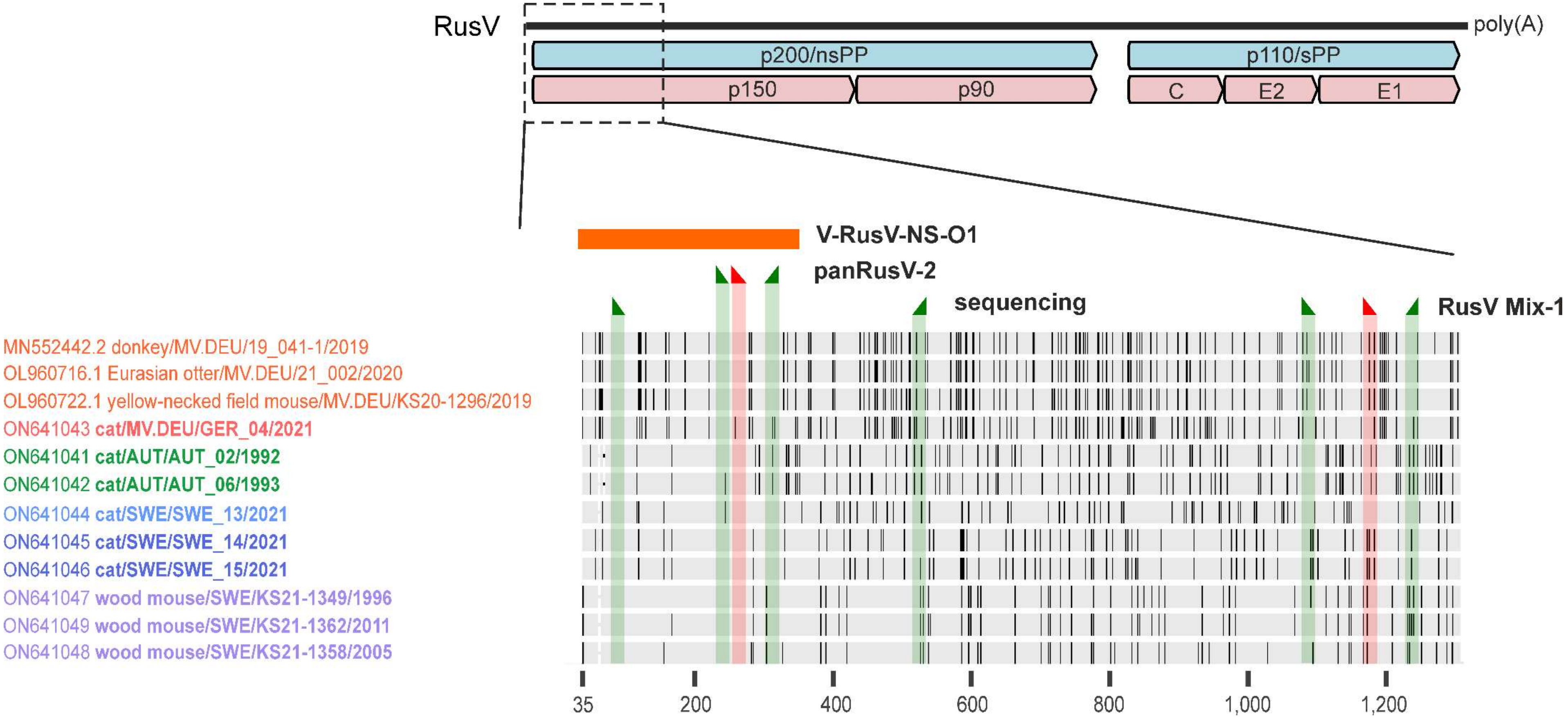

Supplementary Figure 3. Position of primers and probes used for rustrela virus (RusV) RNA detection and sequencing. An alignment of representative RusV sequences from Germany, Sweden, and Austria was used to visualize nucleotide mismatches in each strain (black bars) to the consensus sequence. Only the 5' end, comprising the 5' UTR and parts of the p200 non-structural polyprotein (nsPP) encoding sequence of the RusV genomes, is shown. The numbering refers to the respective genome positions in the alignment. The positions of the primers and probes of previously published RT-qPCR assays RusV Mix-1[1], and panRusV-2 designed in this study, as well as those primers used for Sanger sequencing are highlighted as green (primers) and red (TaqMan probes) triangles. The orange box indicates the stretch of the consensus of the Swedish sequence type that was used to design RNAscope probe V-RusV-NS-O1.

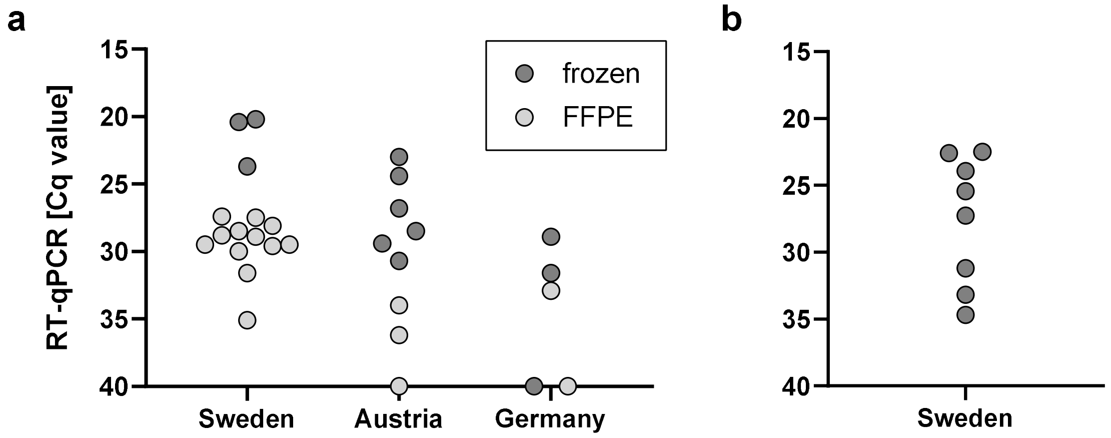

Supplementary Figure 4. Detection of rustrela virus (RusV) RNA by panRusV RT-qPCR in brain samples. (**a**) Cats meeting the inclusion criteria for 'staggering disease'. (**b**) Free-ranging wood mice (*Apodemus sylvaticus*) collected during monitoring studies in Grimsö (Örebro county) in Sweden. Only animals with positive results are depicted for this panel.

Cq: cycle of quantification; FFPE: formalin-fixed paraffin-embedded tissue.

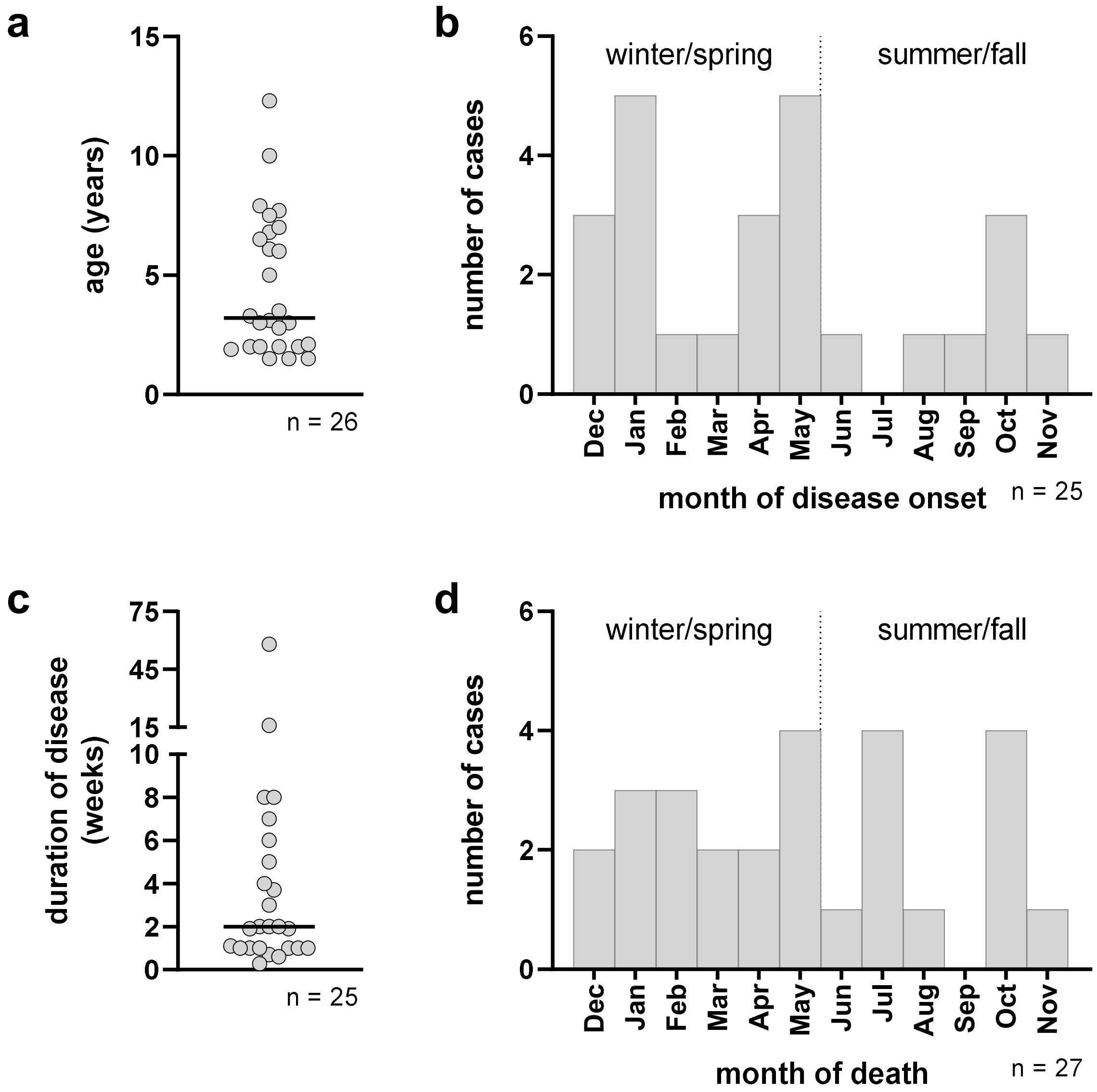

Supplementary Figure 5. Demographic data of 27 rustrela virus (RusV)-infected cats from Sweden, Austria, and Germany. (a) Age and (c) duration of disease as reported by the submitting veterinarian. (b, d) Seasonal distribution of disease onset and month of death of RusV-infected cats. The age, month of disease onset, and duration of disease were unknown for one, two, and two cats, respectively.

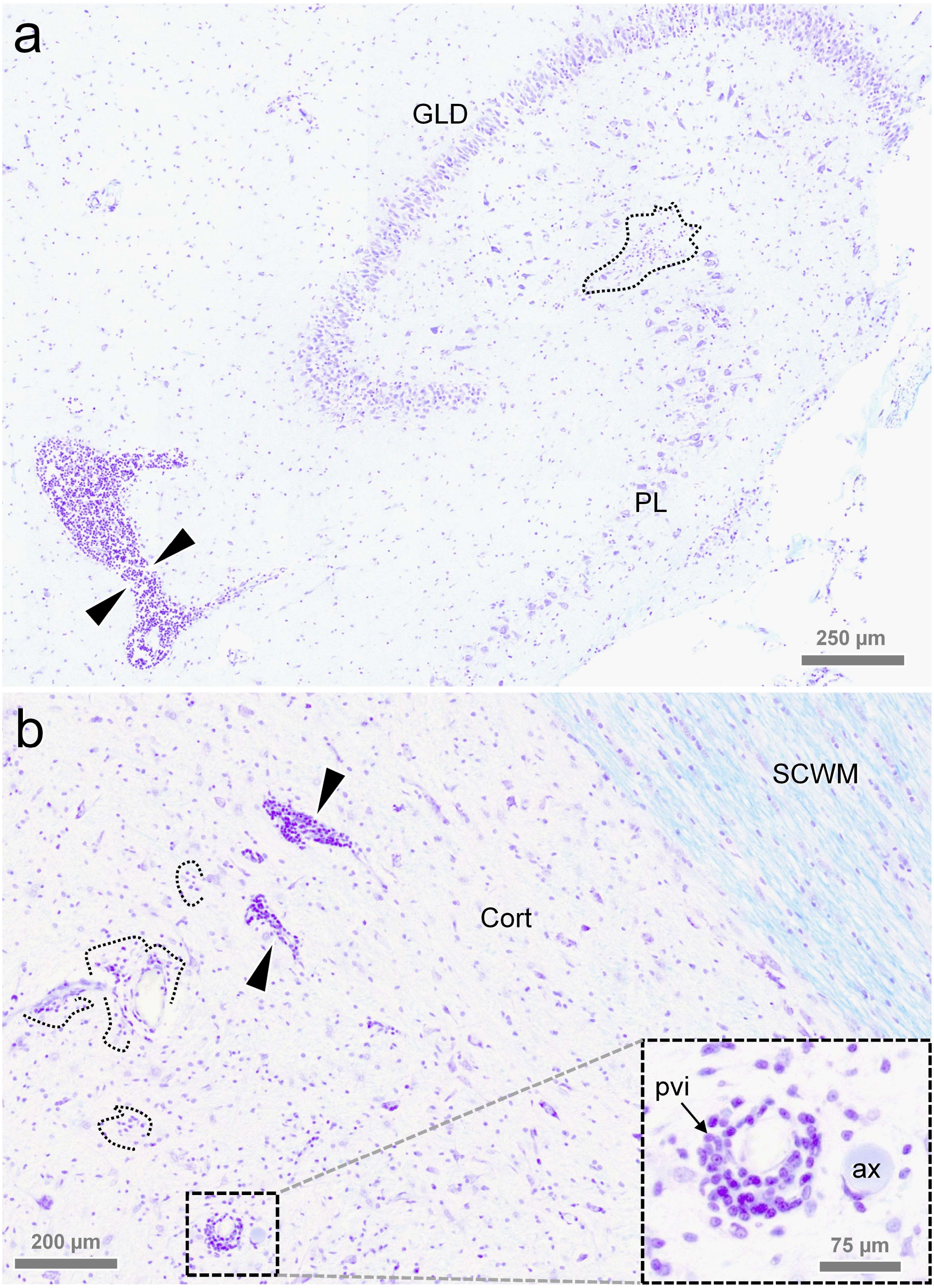

Supplementary Figure 6. Inflammation and cytopathological features on Luxol Fast Blue-Cresyl-Echt Violet stained slides. A selection of cases underwent Luxol Fast Blue-Cresyl- Echt Violet stain according to Klüver-Barrera[4] to compare predominance of grey matter versus white matter affection and to highlight neuronal cytopathology. Leukocytic infiltrates were more prominent in

grey matter (**a**, **b**: cort) compared to white matter (**b**: SCWM). They consist of both well circumscribed perivascular cuffs (**a**, **b**: arrowheads) and ill-defined infiltrates mingling with glial proliferates (**a**, **b**: dashed lines). At high magnification (**b inlet**: pvi) perivascular infiltrates are composed of mononuclear cells comprising mainly lymphocytes and histiocytes. Affected neurons show hyaline axonal spheroids (**b inlet**: ax), occasional shrinkage, displaced and mottled Nissl substance (not shown).

Anatomical landmarks: Cort: cortex; GLD: granule cell layer of dentate gyrus; PL: pyramidal cell layer of hippocampus; SCWM: subcortical white matter. Source: (**a**) cat SWE_07, (**b**) cat SWE_12. Representative images of RusV-infected cats are presented. A selection of four staggering disease cases and seven control cats (five non-encephalitic, two encephalitic)) from Sweden and Germany underwent this staining.

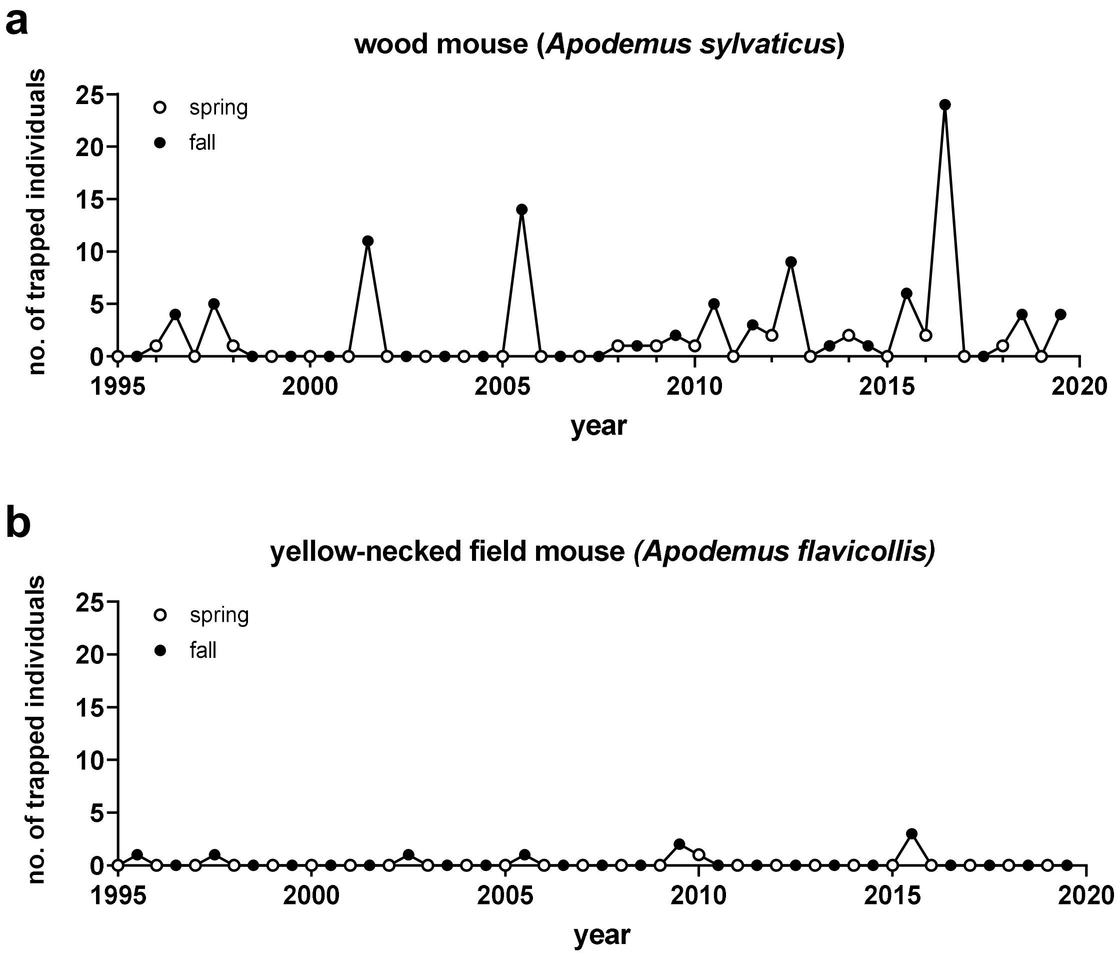

Supplementary Figure 7. Number of trapped rodents (*Apodemus* spp.) from Grimsö, Sweden, examined during this study. The animals had been collected as part of the Swedish Environmental Monitoring Program of Small Rodents[5] during 2,610 to 2,895 trap nights per year and season.

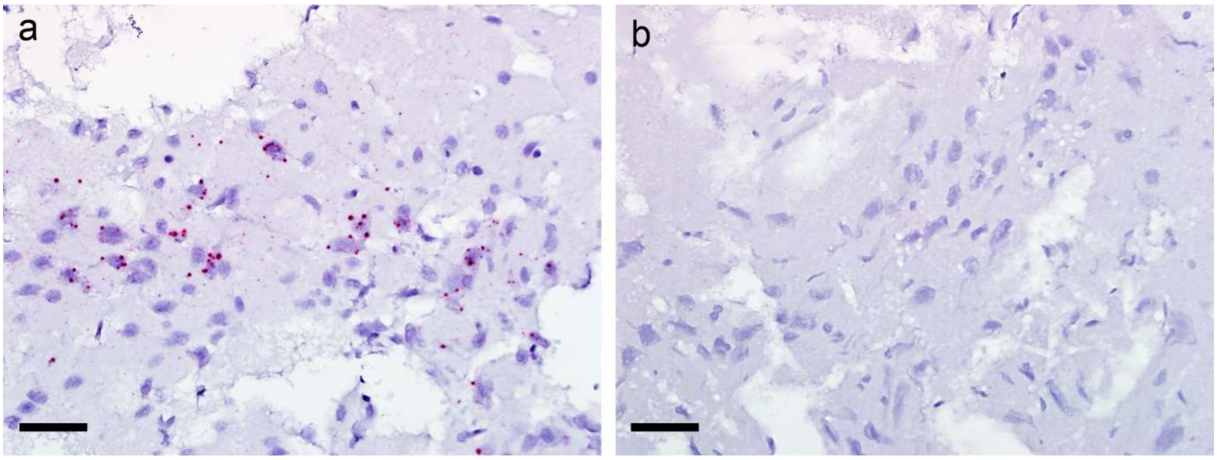

Supplementary Figure 8. Localization of rustrela virus (RusV) RNA by RNAscope *in-situ* hybridization in the brain of wood mice (*Apodemus sylvaticus*). (**a**) Abundant, predominately spherical reaction products in neurons and neuropil of the cerebral cortex of an RT-qPCR-positive wood mouse. (**b**) No reactivity in an RT-qPCR-negative wood mouse. Despite considerable freezing artefacts, the presence or absence of specific reactivity was clearly recognizable in all tested individuals. Scale bars = 40 µm. Representative images are presented. Brain sections of only four RusV-infected and one non-infected wood mice, as confirmed by specific RT-qPCR, possessed sufficient sample quality for ISH analysis. All four PCR-positive mice provided positive ISH results.

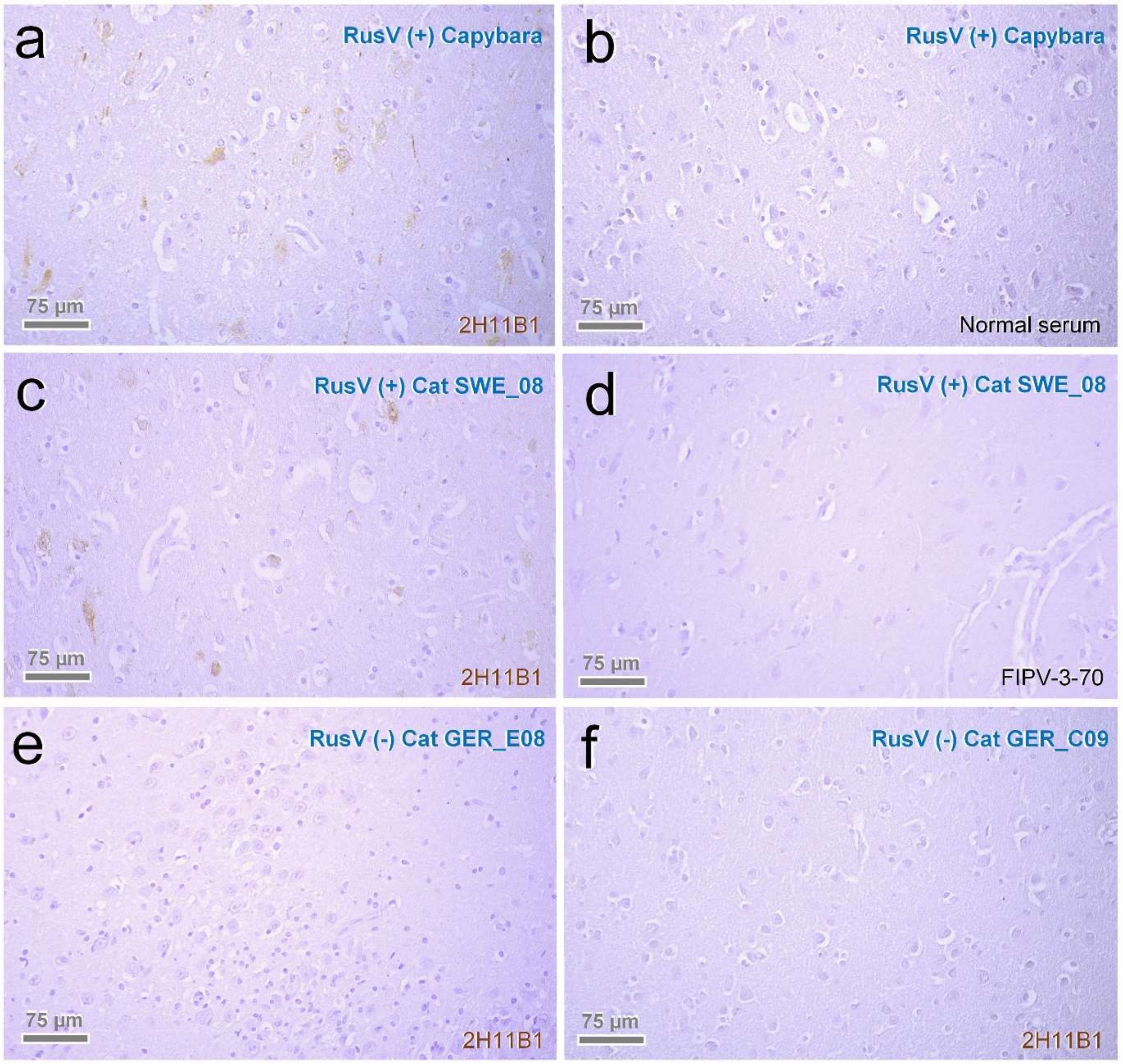

Supplementary Figure 8. Control reactions of immunohistochemistry (IHC). PCR-confirmed RusV-positive capybara brain was used as positive control for RusV IHC using mouse monoclonal antibody 2H11B1 targeting the RusV capsid protein (a). To assure specific staining of the capybara brain by 2H11B1, these slides were flanked by control slides with replacement of 2H11B1 by normal horse serum (b). For cats showing positive 2H11B1 signal (c), unspecific binding of murine antibodies was evaluated by immersion with an irrelevant murine primary antibody (FIPV-3-70) (d). To further confirm the specificity of antibody 2H11B1, brains of PCR-negative encephalitic (e) and non-encephalitic (f) control cats were analysed by RusV IHC (a-f). Counterstain: haematoxylin. Representative images of RusV-infected cats are presented. All case and control cats (n=29 each) were analysed. Results of IHC analysis are presented in Table 1 and Figure 2.

Supplementary Table 1. Primers and probes used for bornavirus and rustrela virus (RusV) RNA detection, internal control RNA amplification, and Sanger sequencing.

Assay	Primer/Probe name	Sequence (5' to 3')	Reference
panBorna v7.2	Borna-1319-F	CGCGACCMTCGAGYCTRGT	Schlottau *et al.*[6]
	Borna-1471.2-FAM	FAM-AAGAAYCCHTCCATGATCTCMGAYCMAGA-BHQ1	Schlottau *et al.*[6]
	Borna-1529-R	GACARCTGYTCCCTTCCKGT	Schlottau *et al.*[6]
BoDV-1 Mix-1	BoDV-1_1258+	TAGTYAGGAGGCTCAATGGCA	Schlottau *et al.*[6]
	BoDV-1_1316_FAM	FAM-AAGAAGATCCCCAGACACTACGACG-BHQ1	Schlottau *et al.*[6]
	BoDV-1_1419-	GTCCYTCAGGAGCTGGTC	Schlottau *et al.*[6]
RusV Mix-1	RusV_1072+	CGAGCGTGTCTACAAGTTCA	Bennett *et al.*[1]
	RusV_1161_P	FAM-CCGAGGAGGACGCCCTGTGC-BHQ1	Bennett *et al.*[1]
	RusV_1237-	GACCATGATGTTGGCGAGG	Bennett *et al.*[1]
panRusV-2	RusV_234+	CCCCGTGTTCCTAGGCAC	this study
	RusV_256_P	FAM-GTGAGCGACCACCCAGCACTCCA-BHQ1	this study
	RusV_323-	TCGCCCCATTCWACCCAATT	this study
eGFP mix 1	EGFP-1-F	GACCACTACCAGCAGAACAC	Hoffmann *et al.*[7]
	EGFP-Probe1_HEX	HEX-AGCACCCAGTCCGCCCTGAGCA-BHQ1	Hoffmann *et al.*[7]
	EGFP-2-R	GAACTCCAGCAGGACCATG	Hoffmann *et al.*[7]
conventional RusV RT-PCR & Sanger sequencing	RusV_80+	GTCGAGGAGCAGATAAGCCC	this study
	RusV_528-	AGCGCCGGGTCYGTRACAAC	this study

Supplementary Table 2. Demographic data on cats with ‘staggering disease’.

Cat ID	Cat ID (submitter)	Month of death	Disease duration [weeks]	Location of origin	Age [years]	Sex [a]	Outdoor access
SWE_01	010/17	01-2017	1	Skutskär	6.8	MN	yes
SWE_02	021/17	01-2017	3.7	Rimbo	1.5	MN	yes
SWE_03	036/17	02-2017	2	Bålsta	7.7	MN	yes
SWE_04	037/17	02-2017	1	Uppsala	6.1	FN	yes
SWE_05	0201/17	05-2017	1.9	Krylbo	3.1	MN	yes
SWE_06	0202/17	05-2017	0.7	Gävle	7.9	MN	yes
SWE_07	0313/18	10-2018	2	Tärnsjö	6.5	MN	yes
SWE_08	013/19	02-2019	7	Östervåla	3.0	FN	yes
SWE_09	096/19	03-2019	1.9	Alunda	1.9	MN	yes
SWE_10	0167/19	05-2019	1.1	Uppsala	12.3	MN	yes
SWE_11	PAT 6729/19	07-2019	6	Björklinge	7.5	MN	yes
SWE_12	PAT 6755/19	07-2019	8	Uppsala	3.5	MN	yes
SWE_13	21_092-17	04-2021	58	Stockholm	7.0	FN	yes
SWE_14	0305/21	11-2021	1	Vattholma	3.3	MN	yes
SWE_15	0327/21	12-2021	0.6	Uppsala	2.8	MN	yes
AUT_01 [c]	1961/91	10-1991	1	Ollersdorf	2.0	FN	yes
AUT_02 [c]	706/92	04-1992	N/A	N/A	adult	MN	N/A [b]
AUT_03 [c]	1230/92	07-1992	4	Vienna	1.5	MN	yes
AUT_04 [c]	548/93	03-1993	8	Gänserndorf	2.0	MN	yes
AUT_05 [c]	1009/93	06-1993	3	Glinzendorf	2.0	MN	yes
AUT_06 [c]	1533/93	08-1993	1	Prottes	1.5	MN	yes
AUT_07 [c]	1807/93	10-1993	4	Obersiebenbrunn	2.0	M	yes
AUT_08 [c]	1812/93	10-1993	1	Vienna	1.5	M	yes
AUT_09 [c]	2281/93	12-1993	N/A	Gänserndorf	1.5	M	yes
GER_01 [d]	S426/17	05-2017	2	Hannover	2.1	MN	yes
GER_02	NP 387/19	07-2019	16	Leipzig	5.0	MN	yes
GER_03	21TRD0484	03-2021	0.1	Stralsund	13.0	FN	N/A
GER_04	21TRD0953	07-2021	5	Usedom	3.0	FN	yes
GER_05	S34/22	01-2022	0.3	Berlin	6.0	F	yes

[a] F: female; M: male; N: neutered

[b] N/A: information not available

[c] Described in detail in previous publications[8, 9].

[d] Described in detail as case 1 in Nessler *et al.*[10].

Supplementary Table 3. Summary of clinical signs, outcome, and histopathological diagnosis in cats with 'staggering disease'.

Cat ID	Clinical signs and outcome	Histological diagnosis
SWE_01	Ataxia, stiff gait, progressive inability to walk and stand, inability to retract claws; reduced/loss of menace response, palpebral, pupillary reflexes, postural (especially front limbs) reactions, withdrawal, panniculus, and perineal reflexes; depression, intermittent panting, obtundation; euthanasia after disease duration of one week	Mild multifocal lymphohistiocytic MEM [a]
SWE_02	Ataxia, slow gait, falling over; reduced postural reactions (especially hind limbs); mild anisocoria, increased vocalization, contact seeking, hyperthermia, hyporexia, weight loss, pneumonia, obtundation; euthanasia after disease duration of 26 days	Moderate multifocal lymphohistiocytic MEM
SWE_03	Ataxia, falling over; reduced menace response and postural reactions; euthanasia after approximately two weeks of disease duration	Moderate multifocal lymphohistiocytic MEM
SWE_04	Ataxia, weakness of front limbs; reduced/loss of menace response, palpebral reflex, postural reactions, and withdrawal reflexes of front limbs; tremor, hyperesthesia at lumbar spine, mild anisocoria, obtundation, hyperthermia, hyporexia; euthanasia after disease duration of six days	Severe multifocal lymphohistiocytic MEM
SWE_05	Ataxia, falling over, weakness, hypermetria, stiff front limbs on flexion, increased muscle tone of all limbs, tremor; reduced/loss of menace response, palpebral reflex, postural reaction, and withdrawal reflexes; increased vocalization, nervous, staring gaze; euthanasia after disease duration of 13 days	Mild to moderate multifocal lymphohistiocytic MEM
SWE_06	Ataxia, falling over; reduced/loss of menace response and postural reactions (especially hind limbs); fecal incontinence, withdrawn behaviour, somnolence, obtundation; euthanasia after disease duration of five days	Mild to moderate multifocal lymphohistiocytic MEM
SWE_07	Ataxia, wide-legged gait (especially hind limbs), difficulty in jumping, inability to retract claws; reduced panniculus and perianal reflexes; hyperesthesia at lumbar area and tail, kyphosis, hyperthermia, hyporexia, obtundation, cystitis; euthanasia after disease duration of approximately two weeks	Severe multifocal lymphohistiocytic MEM
SWE_08	Ataxia/paresis, weakness of hind limbs, difficulty in jumping, inability to retract claws at hind limbs, hyperesthesia at lumbar back, pelvis, tail and caudal abdomen; increased vocalization, withdrawn behaviour, obtundation, initial hyperthermia, hyporexia, weight loss, pollakisuria, stranguria; euthanasia after disease duration of approximately seven weeks	Severe multifocal lymphohistiocytic MEM
SWE_09	Ataxia, stiff gait, difficulty in jumping; loss of menace response; increased vocalization, contact seeking, hyperthermia, hyporexia, weight loss; euthanasia after disease duration of 13 days	Severe multifocal lymphohistiocytic MEM
SWE_10	Ataxia, stiff gait, intermittent rigidity of right limbs; reduced/loss of menace response, palpebral reflex, and postural reflexes; disorientation, staring gaze, obtundation, hyporexia, weight loss; euthanasia after disease duration of eight days	Moderate multifocal lymphohistiocytic MEM

(continued on next page)

(continued from previous page)

SWE_11	Ataxia, increased muscle tone, left head tilt, progressive lateralized signs, tremor, generalized seizures; euthanasia after disease duration of approximately six weeks	Mild multifocal lymphohistiocytic MEM
SWE_12	Ataxia, difficulties in jumping, falling over, paresis, weakness, somnolence, difficulties in drinking; euthanasia after disease duration of approximately eight weeks	Moderate multifocal lymphohistiocytic MEM
SWE_13	Ataxia, increased muscle tone, tetraparesis; reduced/loss of menace response and postural reactions; depression; euthanasia after disease duration of 58 weeks	Moderate multifocal lymphohistiocytic MEM
SWE_14	Ataxia of hindlimbs, wide-legged gait, falling over, paresis; reduced/loss of cranial nerve reflexes and postural reactions; increased salivation, obtundation; euthanasia after disease duration of seven days	Moderate multifocal lymphohistiocytic MEM
SWE_15	Ataxia, wide-legged gait, fasciculations, behavioural changes, increased vocalization; euthanasia after disease duration of four days	Severe multifocal lymphohistiocytic ME [b]
AUT_01 [c]	Ataxia of one hind limb progressing to hemiparesis; euthanasia after disease duration of one week	Severe multifocal lymphohistiocytic MEM
AUT_02 [c]	Paralysis (no further records)	Moderate multifocal lymphohistiocytic MEM
AUT_03 [c]	Ataxia of hind limbs leading to flaccid paralysis; obtundation, hyporexia, hyperthermia; euthanasia after disease duration of four weeks	Severe multifocal lymphohistiocytic MEM
AUT_04 [c]	Ataxia of hind limbs, inability to jump and walk stairs, inability to retract claws; mydriasis, somnolence; euthanasia after disease duration of eight weeks	Severe multifocal lymphohistiocytic MEM
AUT_05 [c]	Ataxia and paralysis of hind limbs, circling; inability to retract claws, inappetence, somnolence, recumbency, hyperthermia; euthanasia after disease duration of three weeks	Moderate multifocal lymphohistiocytic MEM
AUT_06 [c]	Ataxia, falling over, recumbency with spastic tetraparesis, opisthotonus; euthanasia after disease duration of one week	Mild to moderate multifocal lymphohistiocytic MEM
AUT_07 [c]	Stiff gait, inability to jump, falling over, affectionate behaviour; euthanasia after disease duration of four weeks	Moderate multifocal lymphohistiocytic MEM
AUT_08 [c]	Ataxia, spastic paresis of hind limbs; euthanasia after disease duration of one week	Moderate multifocal lymphohistiocytic MEM
AUT_09 [c]	Stiff gait, inability to jump, falling over; euthanasia (disease duration not recorded)	Severe multifocal lymphohistiocytic MEM

(continued on next page)

(continued from previous page)

GER_01 [d]	Compulsive pacing, increased muscle tone, whole body tremor, hyperthermia, hyporexia, disorientation, obtundation; euthanasia after disease duration of two weeks	Moderate multifocal lympho-histiocytic and plasmacytic MEM and vasculitis
GER_02	Reduced postural reactions of all four limbs; absent menace response on both eyes and absent physiological nystagmus, obtundation, disorientation; euthanasia after disease duration of 16 weeks	Moderate multifocal lymphohistiocytic MEM
GER_03 [e]	Ataxia, salivation, inability to drink, aggression; euthanasia after disease duration of one day	Mild to moderate multifocal lymphohistiocytic ME [b]
GER_04	Ataxia of hind limbs, aggression, hyperthermia, apathy, hyporexia; euthanasia after disease duration of five weeks	Severe multifocal lymphohistiocytic MEM
GER_05	Stupor, spasm, behavioural changes (aggression); euthanasia after disease duration of two days	Moderate multifocal lymphohistiocytic ME [b]

[a] MEM: meningoencephalomyelitis

[b] ME: meningoencephalitis; spinal cord not available for histologic evaluation

[c] Described in detail in previous publications[8, 9].

[d] Described in detail as case 1 in Nessler *et al.*[10].

[e] Cat GER_03 tested negative for RusV infection in all employed assays.

Supplementary Table 4. Demographic data on control cats included in this study.

Cat ID	Cat ID (submitter)	Month of death	Diagnosis	Location of origin	Age [years]	Sex [a]	Outdoor access	CNS tissue for RNA extraction
GER_E01 [b]	S1110/17	11-2017	mild to severe multifocal lymphohistiocytic ME [c,d]	Hemmingen	17.8	FN	yes	FFPE
GER_E02 [b]	S1179/17	12-2017	non-suppurative myelitis [d]	Höxter	2.5	F	yes	FFPE
GER_E03 [b]	S211/19	02-2019	mild to moderate multifocal lymphohistiocytic ME [d]	Hannover	8.4	MN	yes	FFPE
GER_E04	S836/18	11-2018	FCoV-associated ME-ventriculitis [e]	Munich	1.0	MN	yes	frozen
GER_E05	S963/18	12-2018	FCoV-associated ME-ventriculitis [e]	Memmingen	10.0	F	yes	frozen
GER_E06	S321/19	06-2019	FCoV-associated ME-ventriculitis [e]	Neumarkt	9.0	F	yes	frozen
GER_E07	NP 166/20	03-2020	moderate focal lymphohistiocytic ME [d]	Hausham	11.0	MN	yes	FFPE
GER_E08	NP 001/21	12-2020	severe limbic encephalitis	Vohenstrauß	adult	MN	yes	FFPE
SWE_C01	0297/21	11-2021	chronic purulent rhinosinusitis	Rimbo	17.3	MN	yes	frozen
SWE_C02	0304/21	11-2021	hypertrophic cardiomyopathy, aortic thromboembolism	Borlänge	5.5	FN	yes	frozen
SWE_C03	0308/21	11-2021	lymphoma	Tobo	3.5	FN	no	frozen
SWE_C04	0311/21	12-2021	cardiomyopathy, chronic heart failure	Uppsala	7.3	MN	no	frozen
SWE_C05	0346/21	12-2021	lymphoma	Gävle	11.4	FN	N/A [f]	frozen
SWE_C06	0348/21	12-2021	intestinal invagination	Avesta	5.8	MN	no	frozen
SWE_C07	22-pat159	01-2022	acute lethal trauma	Solna	1.5	MN	yes	frozen
AUT_C01	1117/21	11-2021	cardial failure, nephritis	Vienna	6.0	FN	N/A	frozen
AUT_C02	1121/21	11-2021	myocardial fibrosis	Vienna	8.0	MN	yes	frozen
AUT_C03	1122/21	11-2021	ruptured haemangiosarcoma	Vienna	9.0	FN	N/A	frozen
AUT_C04	1123/21	11-2021	lymphoma	Dürnkrut	9.0	MN	yes	frozen
AUT_C05	1168/21	12-2021	chronic renal failure	Vienna	13.0	FN	yes	frozen
GER_C01	S690/18	09-2018	hydrocephalus	Munich	1.0	MN	N/A	frozen
GER_C02	S693/18	09-2018	traumatic brain injury	Winzer	0.1	F	no	frozen
GER_C03	S326/19	06-2019	brain tumor	Munich	10.0	M	N/A	frozen
GER_C04	S388/19	07-2019	cardiomyopathy, hepatopathy	Munich	9.5	MN	N/A	frozen
GER_C05	S438/19	08-2019	enteritis	Kirchroth	18.0	MN	yes	frozen
GER_C06	S445/19	08-2019	hippocampal sclerosis	Bad Tölz	7.0	F	yes	frozen
GER_C07	S390/20	02-2020	septic encephalopathy	Kempten	9.0	F	yes	frozen
GER_C08	S477/20	08-2020	hydrocephalus	N/A	6.0	F	N/A	frozen
GER_C09	S564/20	09-2020	traumatic brain injury	Dietramszell	11.0	FN	yes	frozen

[a] F: female; M: male; N: neutered

[b] Described in detail as cases 2 to 4 in Nessler *et al.*[10]

[c] ME: meningoencephalitis
[d] Non-suppurative meningoencephalitis or myelitis not matching the criteria of 'staggering disease' due to multifocal neuronal and white matter necrosis and hypertrophy of endothelial cells (GER_E01), diffuse neuronal necrosis and gemistocytosis (GER_E02), focal spinal cord white matter degeneration with associated infiltration of mononuclear inflammatory cells (GER_E03) and focal extensive, white matter-predominant, lymphohistiocytic encephalitis without neuronal changes or evidence of neuronal tropism (GER_E07),
[e] FCoV: feline coronavirus
[f] N/A: information not available

Supplementary References

1. Bennett AJ*, et al.* Relatives of rubella virus in diverse mammals. *Nature* **586**, 424-428 (2020).

2. Pfaff F*, et al.* Revisiting rustrela virus: new cases of encephalitis and a solution to the capsid enigma. *Microbiol Spectr* **10**, e0010322 (2022).

3. Bennett AJ*, et al.* Author Correction: Relatives of rubella virus in diverse mammals. *Nature* **588**, E2 (2020).

4. Klüver H, Barrera E. A method for the combined staining of cells and fibers in the nervous system. *J Neuropathol Exp Neurol* **12**, 400-403 (1953).

5. Ecke F, Hörnfeldt B. Miljöövervakning av smågnagare. Preprint at http://www.slu.se/mo-smagnagare (2022).

6. Schlottau K*, et al.* Fatal encephalitic Borna disease virus 1 in solid-organ transplant recipients. *The New England journal of medicine* **379**, 1377-1379 (2018).

7. Hoffmann B, Depner K, Schirrmeier H, Beer M. A universal heterologous internal control system for duplex real-time RT-PCR assays used in a detection system for pestiviruses. *J Virol Methods* **136**, 200-209 (2006).

8. Nowotny N, Weissenböck H. Description of feline nonsuppurative meningoencephalomyelitis ("staggering disease") and studies of its etiology. *Journal of clinical microbiology* **33**, 1668-1669 (1995).

9. Weissenböck H, Nowotny N, Zoher J. Feline Meningoencephalomyelitis ("Staggering Disease") in Österreich. *Wien Tierärztl Mschr* **81**, 195-201 (1994).

10. Nessler J*, et al.* Meningoencephalomyelitis of unknown origin in cats: A case series describing clinical and pathological findings. *Front Vet Sci* **7**, 291 (2020).
